# Supplementary material for: Axial localization and tracking of self-interference nanoparticles by lateral point spread functions
Source: Nat Commun. 2021 Apr 1;12:2019. doi: 10.1038/s41467-021-22283-0 (PMC8016974; doi:10.1038/s41467-021-22283-0)
Supplement: Supplementary file 1 — Supplementary Information [file 41467_2021_22283_MOESM1_ESM.docx]

Supplementary information

Axial Localization and Tracking of Self-interference Nanoparticles by Lateral Point Spread Functions

Yongtao Liu^1^, Zhiguang Zhou^1^, Fan Wang^1,2*^, Günter Kewes^3^, Shihui Wen^1^, Sven Burger^4^, Majid Ebrahimi Wakiani^1,5^, Peng Xi^6^, Jiong Yang^7^, Xusan Yang^6,8^, Oliver Benson^3*^, Dayong Jin,^1,9*^

^1^Institute for Biomedical Materials and Devices (IBMD), Faculty of Science, University of Technology Sydney, NSW 2007, Australia

^2^School of Electrical and Data Engineering, Faculty of Engineering and Information Technology, University of Technology Sydney, Ultimo 2007, Australia

^3^AG Nanooptik, Institut für Physik & IRIS Adlershof, Humboldt Universität zu Berlin Newtonstraße 15, 12489 Berlin, Germany

^4^JCMwave GmbH, Bolivarallee 22, 14050 Berlin, Germany; Zuse Institute Berlin, Takustraße 7, 14195 Berlin, Germany

^5^School of Biomedical Engineering, Faculty of Science, University of Technology Sydney, NSW 2007, Australia

^6^Department of Biomedical Engineering, College of Engineering, Peking University, Beijing 100871, China

^7^School of Chemical Engineering, University of New South Wales (UNSW), Sydney Campus, NSW 2052 Australia

^8^School of Applied and Engineering Physics, Cornell University, Ithaca 14853, USA

^9^UTS-SUStech Joint Research Centre for Biomedical Materials & Devices, Department of Biomedical Engineering, Southern University of Science and Technology, Shenzhen, Guangdong 518055, P. R. China

These authors contributed equally: Yongtao Liu, Zhiguang Zhou, Fan Wang

^*^Correspondence to [fan.wang@uts.edu.au](mailto:fan.wang@uts.edu.au); [oliver.benson@physik.hu-berlin.de](mailto:oliver.benson@physik.hu-berlin.de); [dayong.jin@uts.edu.au](mailto:dayong.jin@uts.edu.au);

**Supplementary Note 1: Bandwidth requirement for the self-interference**

To achieve efficient interference around the nanoparticle, a narrowband emission is required. In the upconverting system, the emission is from the atomic transition. There is a direct relation between the coherence time $\tau_{c}$, the coherence length $l_{c}$and the spectral width $\Delta\omega$. Supplementary Figure 1 shows the emission spectrum from a typical UCNP. Here we assume each of dipoles inside a UCNP provides the same emission spectrum. Then the FWHM of the emission peak with angular frequency axis ($\Delta\omega$) can be measured from Supplementary Figure 1b. The coherence length can be estimated by:

$l_{c}=c*\tau_{c}/n=c/(n\Delta\omega)$ (1)

here *c* is the speed of light, *n* is the refractive index of the medium. The calculated coherence length for 455 nm and 800 nm are 3.1 μm and 2.7 μm, respectively. In this work, our *z-*axis viewing range is below 500 nm, which is within the coherence length for both 455 nm and 800 nm.

For emitters with broadband emission, such as dye molecules, a narrowband filter is required to maintain the coherence length above 500 nm. For this case, the bandwidth of the filter is required to be narrow than 44 nm for the central wavelength of 455 nm, according to below equations.

$\Delta\omega=\frac{2\pi c\Delta\lambda}{\lambda^{2}}=\frac{c}{nl_{c}}$ (2)

$\Delta\lambda=\frac{\lambda^{2}}{2\pi nl_{c}}$ (3)

**
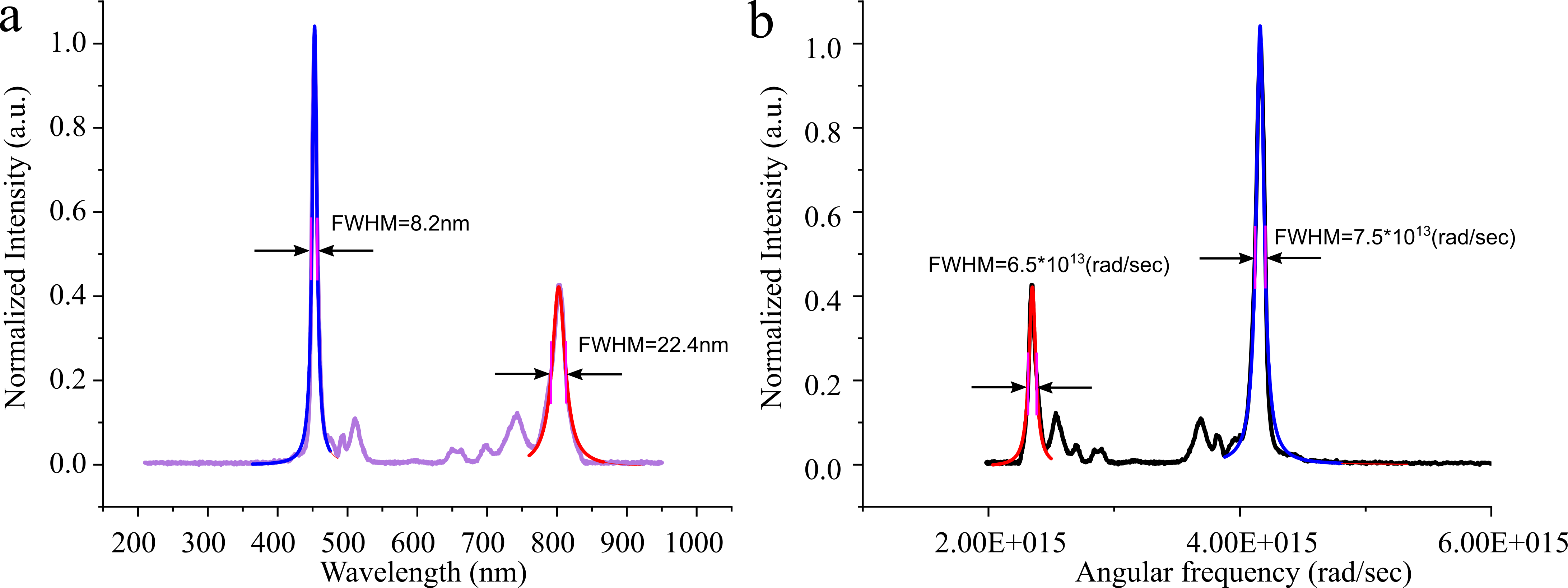
**

**Supplementary Figure 1.** Emission spectra from UCNPs. The emission spectra from a single UCNP on a glass substrate with the horizontal axis as (a) wavelength and (b) angular frequency.

**Supplementary Note 2: Schematic of optical setup, materials, and sample fabrication**

1. *Sample fabrication and preparation*

The mirror used in our experiment is a custom-made (ANFF, Australia), first-surface mirror, using a sliced silicon wafer as a base. A silver film about 200 nm is sputtered on a silicon wafer, and then the SiO_2_ layer is deposited as a spacer by thermal evaporation. In this experiment, a series of spacer thicknesses was prepared (50 nm, 75 nm, 100 nm, 125 nm, 150 nm, 175 nm, 200 nm, 250 nm, 300 nm, 500 nm). The AFM characterized values are 55.1 nm, 87.3 nm, 117.1 nm, 138.4 nm, 157 nm, 170.1 nm, 198.2 nm, 251 nm, 310.4 nm and 467.4 nm. In the main text and the simulation, the distance between the particle and mirror is the spacer thickness plus the effective radius of the UCNP (16.5 nm). Hence in this work, the characterized spacings between the particle’s centre and mirror surface are 71.6 nm, 103.8 nm, 133.6 nm, 154.9 nm, 173.5 nm, 186.6 nm, 214.7 nm, 267.5 nm, 326.9 nm, and 483.9 nm.

During the experiment, the UCNPs are dispersed on the top of mirror samples by the dip-casting method, which provides a better distribution of individual nanoparticles as compared with the drop-casting method. In this dip-casting method, we first dilute the concentration of UCNPs to 0.01 mg/ml. Then, we took 2 ml from the solution and placed them into a shallow dish. Finally, we used a tweezer to hold the mirror, then, we dipped it into the dish, took it out, and left it for drying. A schematic of the sample is shown in Supplementary Figure 3. To avoid the refractive mismatch, we use objective oil to fill the gap between SiO_2_ and the top cover glass. The sample of the UCNP in glycerol solution was prepared in a similar way. UCNPs coated with home-made polymer were purified three times and then diluted into 0.01 mg/ml. Then, the solution was added to 99% Glycerol. The Glycerol with the UCNPs was diluted into a concentration of 93% with water, then transferred into a home-made micro-chamber. As the last step, the micro-chamber was closed with a cover glass.

1. *Schematic of the optical measurement setup*

Supplementary Figure 4 is the optical system, where a 980 nm laser is focused on the back aperture of the objective lens to generate a wide-field excitation. A CCD camera is used to record the fluorescence image for distance sensing. The objective lens is an oil-immersed lens with NA=1.4. The method is valid for an objective lens with other NA values. Supplementary Figure 5 shows the simulated far-field distribution (or Fourier domain) from a UCNP with 154.9 nm silica layer, the collection ability of the system is labelled by a black dashed line. A smaller NA will decrease the collecting angle and the resultant collecting range in the Fourier domain, which modulates the collected amplitudes of emission for dipoles along each direction (x, y, and z). Therefore, a calibration process (e.g. in Figure 3) is required for other NA values. When the effective NA decreases to below 0.6, the amplitude from a z dipole became too low to be comparable with amplitudes for x and y dipoles. Therefore, the method is valid for optical systems with effective NA > 0.6.


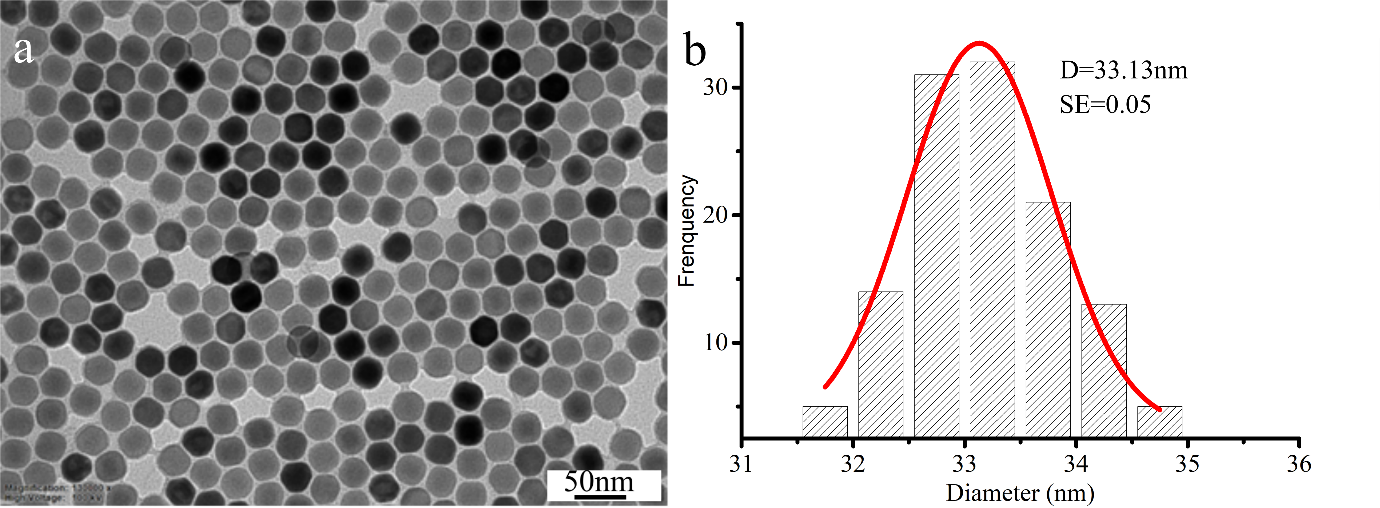


**Supplementary Figure 2.** TEM images of NaYF_4_ nanoparticles. (a) TEM image of the β-NaYF_4_:20%Yb^3+^,8%Tm^3+^ nanoparticles. (b) Size (diameter) distributions of the UCNPs, measured from the TEM image. The mean diameter is 33.13nm, and the standard error is 0.05. The nanoparticles are synthesized by our previously reported method^1^.


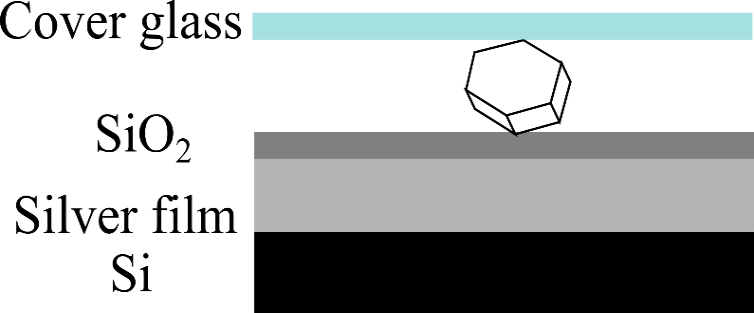


**Supplementary Figure 3**. Schematic structure of the mirror sample.


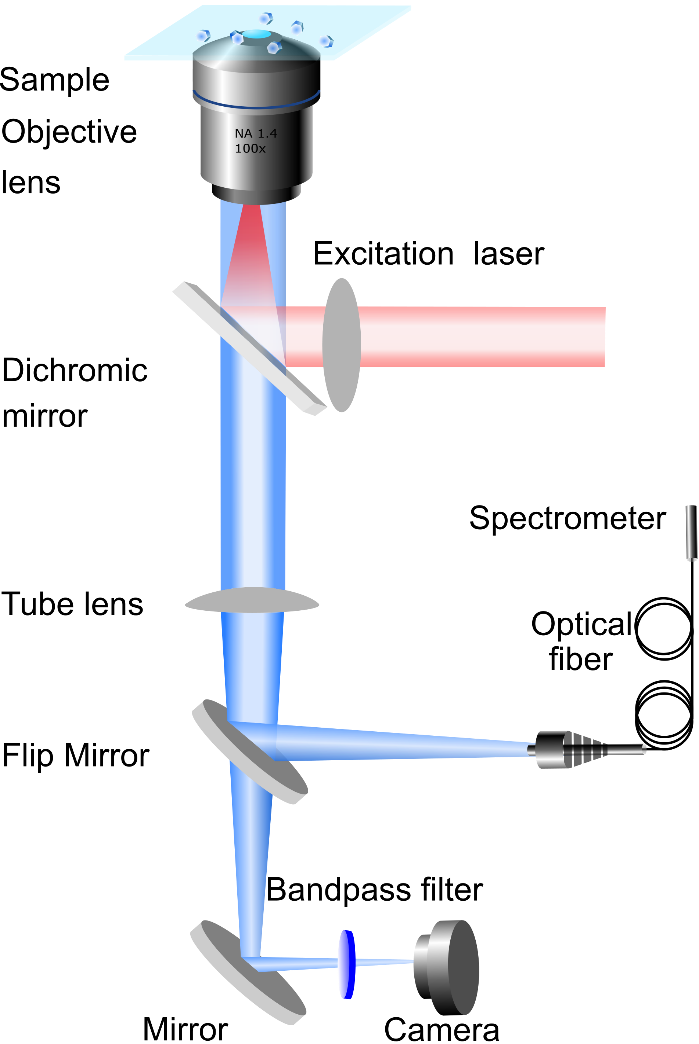


**Supplementary Figure 4.** Schematics of the optical system. Home-made fluorescence imaging system built for single-particle characterization, wide-field imaging, and spectral analysis of the upconversion nanoparticles (UCNPs). For wide-field fluorescence imaging, a 980nm CW diode laser is focused at the back aperture of the objective lens (Olympus, NA 1.4, oil immersion) to generate a wide field excitation field. The imaged is captured by an electron-multiplying CCD (EMCCD) camera. For single-particle characterization and spectral analysis, an optical fibre is used to pick up the emission signal from a single nanoparticle and to send it to a spectrometer.


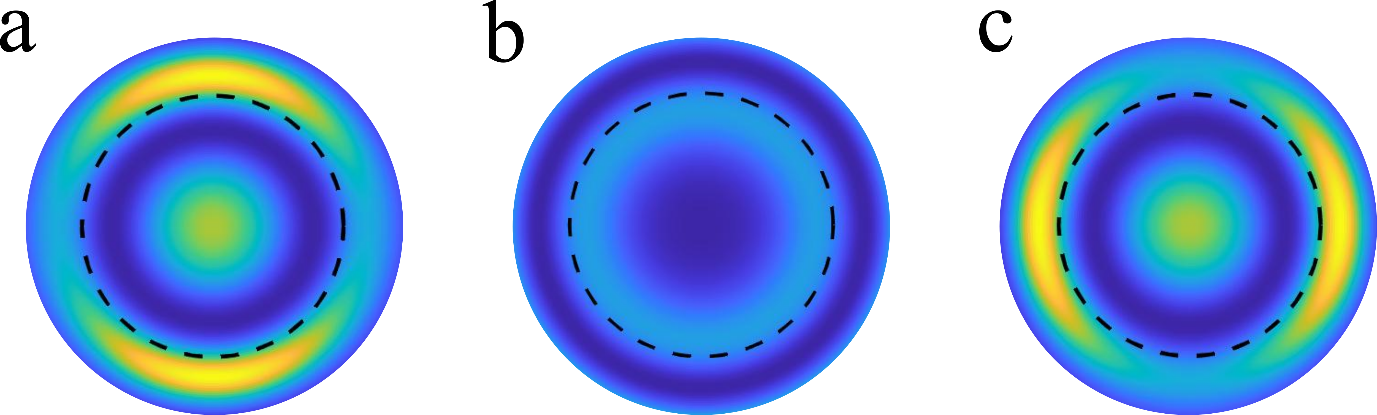


**Supplementary Figure 5.** Simulated far-field emission pattern. Far-field distribution (Fourier domain) intensities for single (a) *x*-dipole, (b) *z*-dipole and (c) *y*-dipole. The dashed line indicates the collection ability of a system with an effective NA=1.01 (estimated from the PSF of the system)

**Supplementary Note 3: Simulations Method**

We determine the through-focus-images of light-emitting nanoparticles placed on top of a layer stack by numerically solving the time-harmonic, linear Maxwell's equations. The light source is modelled by an incoherent sum of three point-like sources (Hertzian dipoles) with orthogonal polarization directions. The use of Hertzian dipole sources naturally includes potentially in- or decreased photon emission rates due to distance-variations of the local density of states near the mirror. We use tabulated material data by Johnson and Christy^2^ for silver, and a constant refractive index of n=1.5 for the SiO_2_ layer, the UCNP host-material and the index matching immersion oil. The geometry of the system is, therefore, a one-dimensional stack of a mirror layer and layers of constant refractive index, and the source is placed at a distance of *R*+*d*_SiO2_ above the silver mirror, where *R* is the half of the UCNP height and *d*_SiO2_ is the thickness of the silica spacer. Image formation through the microscope objective is modelled using Fourier optics^3^. The centre of the UCNP is positioned at the centre of the object plane of the optical system.

For this numerical task, we use a standard PC, and the finite-element method (FEM) based solver JCMsuite. The rotational symmetry of the geometrical setup is taken into account by a self-adaptive expansion in a Fourier series in the rotation angle^4^. For high numerical efficiency in the simulation of the singular fields resulting from point-like sources, a subtraction-field approach is used. This approach relies on avoiding to explicitly compute the singular near field distribution in the vicinity of the dipole source. Instead, the smooth difference field between the known analytical solution to a dipole source in constant background medium and the unknown solution to the problem at hand is computed. For best performance, the difference field is computed in an adaptively chosen domain. Perfectly matched layers (PML) are used to ensure transparent boundary conditions of the computational domain, which typically encloses few cubic wavelengths only. Using a near-field to far-field transformation, the outwards propagating light field is decomposed in a Fourier basis. The Fourier modes are then propagated through JCMsuite’s optical imaging simulator, and through-focus images are exported for comparison and analysis of corresponding experimental results. Near- and far-field for each dipole orientation are calculated separately, and the intensities are summed up at the end, which represents an incoherent interaction of all three linear independent dipole orientations.

Sufficiently high numerical accuracy is ensured by using adaptive approaches in the various discretization steps of the simulation: For a given target accuracy, mesh element size (h) and polynomial order (p) of the FEM ansatz functions are adaptively chosen using a-prior heuristics and a-posteriori error control. Also, the numerical parameters for PML, subtraction field approach, radial Fourier expansion and for the sampling of the continuous Fourier spectrum of outwards propagating light are chosen self-adaptively.

**Supplementary Note 4: Self-interference simulations and experimental result**

1. *Distance induced energy transfer variation*

The plasmon coupling between a UCNP and a metallic film is more complex than the coupling between a dipole and a metallic film, as a UCNP has a multi-level energy upconverting system. Supplementary Figure 6 is the diagram of the up-converting energy transfer process for Yb^3+^ and Tm^3+^ co-doped system. To simplify the energy transferring process, we use a five-energy level model for the simulation, where *n_5_*, *n_4_* and *n_1_* stand for energy levels of ^1^D_2_, ^1^G_4_ and ^3^H_6_ respectively. The *n_3_* is the degeneration of energy levels of ^3^F_2_, ^3^F_3_, and ^3^H_4_, since there is no radiational energy transfer between these levels. For the same reason, the *n_2_* is to represent energy levels of ^3^F_4_, and ^3^H_5_. The excitation photon will be absorbed by sensitizer ions, pumping the electrons from *n_6_* to *n_7_*. Then the energy at *n_7_* is transferred to the emitter for both upconverting and down converting emission. There are three major energy transfer processes simultaneously happening inside a UCNP. The first process is the energy transferring from sensitizers (Yb^3+^) to emitters (Tm^3+^), and the transferring strength is characterized by transferring rate *C* (e.g. *C_1_*, *C_2_*, *C_3_*, *C_4_*). The second process is the carrier decaying on each of excited energy levels. Here we use *W* (e.g. *W_2_*, *W_3_*) to represent the decay rate and use *b* (e.g. *b_54_*, *b_53_*) to represent branching ratio. The third process is the cross-relaxation which plays a critical role for highly doped upconversion nanoparticles^5,6^.


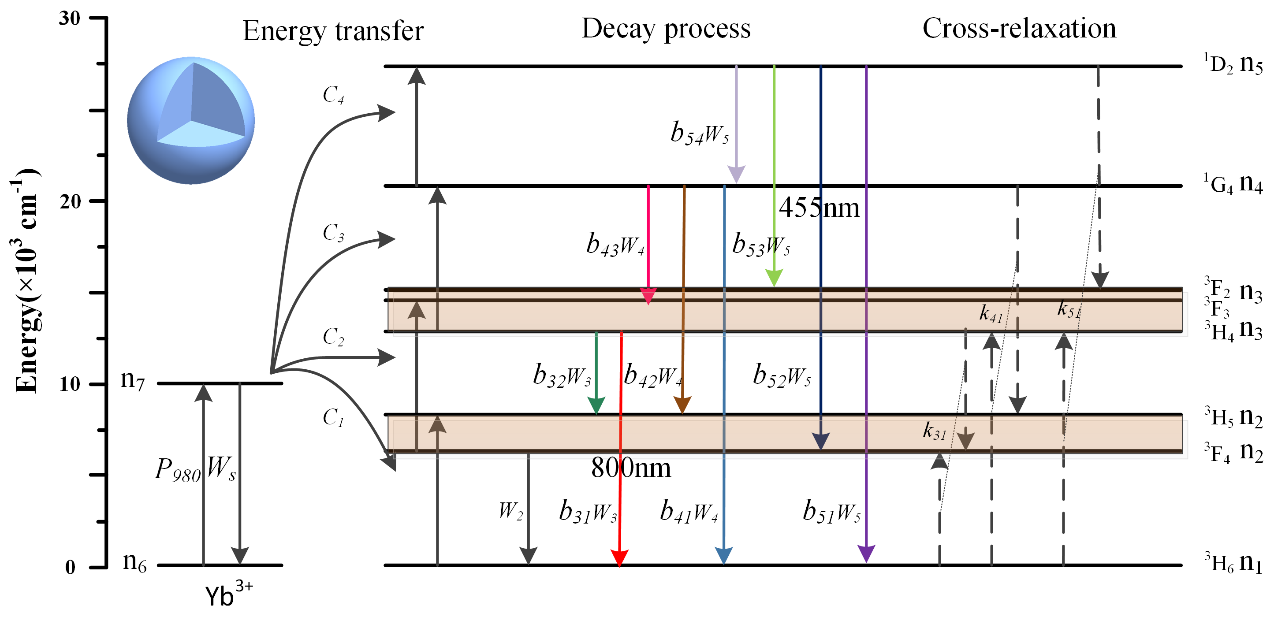


**Supplementary Figure 6**. Diagram of the energy transfer processes of a Yb^3+^ and Tm ^3+^ co-doped upconverting system.

The rate equations to describe the system is shown as:

$$\begin{aligned} \frac{dn_{1}}{dt}={-c}_{1}n_{1}n_{7}+b_{21}w_{2}n_{2}+b_{31}w_{3}n_{3}+b_{41}w_{4}n_{4}+b_{51}w_{5}n_{5}-k_{41}n_{1}n_{4}- \\ k_{31}n_{1}n_{3}-k_{51}n_{1}n_{3}\#\left( 4 \right) \end{aligned}$$

$$\begin{aligned} \frac{dn_{2}}{dt}=c_{1}n_{1}n_{7}-c_{2}n_{2}n_{7}-b_{21}{w_{2}n}_{2}+b_{32}{w_{3}n}_{3}+b_{42}w_{4}n_{4}+b_{52}{w_{5}n}_{5} \\ +k_{41}n_{1}n_{4}+2k_{31}n_{1}n_{3}\#\left( 5 \right) \end{aligned}$$

$$\begin{aligned} \frac{dn_{3}}{dt}=c_{2}n_{2}n_{7}{-c}_{3}n_{3}n_{7}-w_{3}n_{3}+b_{43}{w_{4}n}_{4}+b_{53}{w_{5}n}_{5}+{2k}_{51}n_{5}n_{1}+k_{41}n_{4}n_{1} \\ -k_{31}n_{3}n_{1}\#\left( 6 \right) \end{aligned}$$

$$\begin{aligned} \frac{dn_{4}}{dt}=c_{3}n_{3}n_{7}-c_{4}n_{4}n_{7}-{w_{4}n}_{4}+b_{54}{w_{5}n}_{5}-k_{41}n_{1}n_{4}\#\left( 7 \right) \end{aligned}$$

$$\begin{aligned} \frac{dn_{5}}{dt}=c_{4}n_{4}n_{7}-{w_{5}n}_{5}-k_{51}n_{1}n_{5}\#\left( 8 \right) \end{aligned}$$

$$\begin{aligned} \frac{dn_{7}}{dt}=\frac{P\delta}{h\nu}n_{6}-w_{7}n_{7}-\left( c_{1}n_{1}+c_{2}n_{2}+c_{3}n_{3}+c_{4}n_{4} \right)n_{7}\#\left( 9 \right) \end{aligned}$$

Here $\delta$ is the absorption cross-section, *P* is the excitation power, $\nu$ is the frequency of the excitation photon. The Plasmon enhancement mechanism for Yb^3+^ and Er^3+^ co-doped upconverting system has been developed by Dawei Lu et al.^7^. The mechanism for Yb^3+^ and Tm^3+^ co-doped upconverting system is similar. The metal surface that closes to a UCNP provides both plasmon enhancement and quenching effects. According to Dawei Lu et al. and Christian Clarke et al.^8^, the quenching effect will induce a large reduction in decay time. In our experiment, due to the larger spacing (> 71.6 nm) the quenching effect is minimized, which is proved by the lifetime measurement shown in Supplementary Figure 7. The plasmon enhancement will enhance the absorption cross-section ($\delta$), the energy transferring rate (*C*) and the decay rate (*W*). In our experimental condition, we are using the excitation power density of 0.95 MW/cm^2^, which achieves saturation condition^9^. Hence the enhancing in $\delta$ will not affect the energy transferring process. According to Dawei Lu et al.^7^ and Dung H. T. et al.^10^, when the distance is larger than 0.1$\lambda_{sp}$ the enhancement on energy transferring can be ignored. Here the surface plasmon wavelength $\lambda_{sp}$, at which $\varepsilon_{1}=-Re\left( \varepsilon_{2} \right)$, is 361 nm; $\varepsilon_{1}$ and $\varepsilon_{2}$ are the permittivity’s of SiO_2_ and silver, respectively. Because the distance between UCNP and film is larger than 71.6 nm that > 0.1$\lambda_{sp}$, the enhancement on energy transferring rate can be ignored.


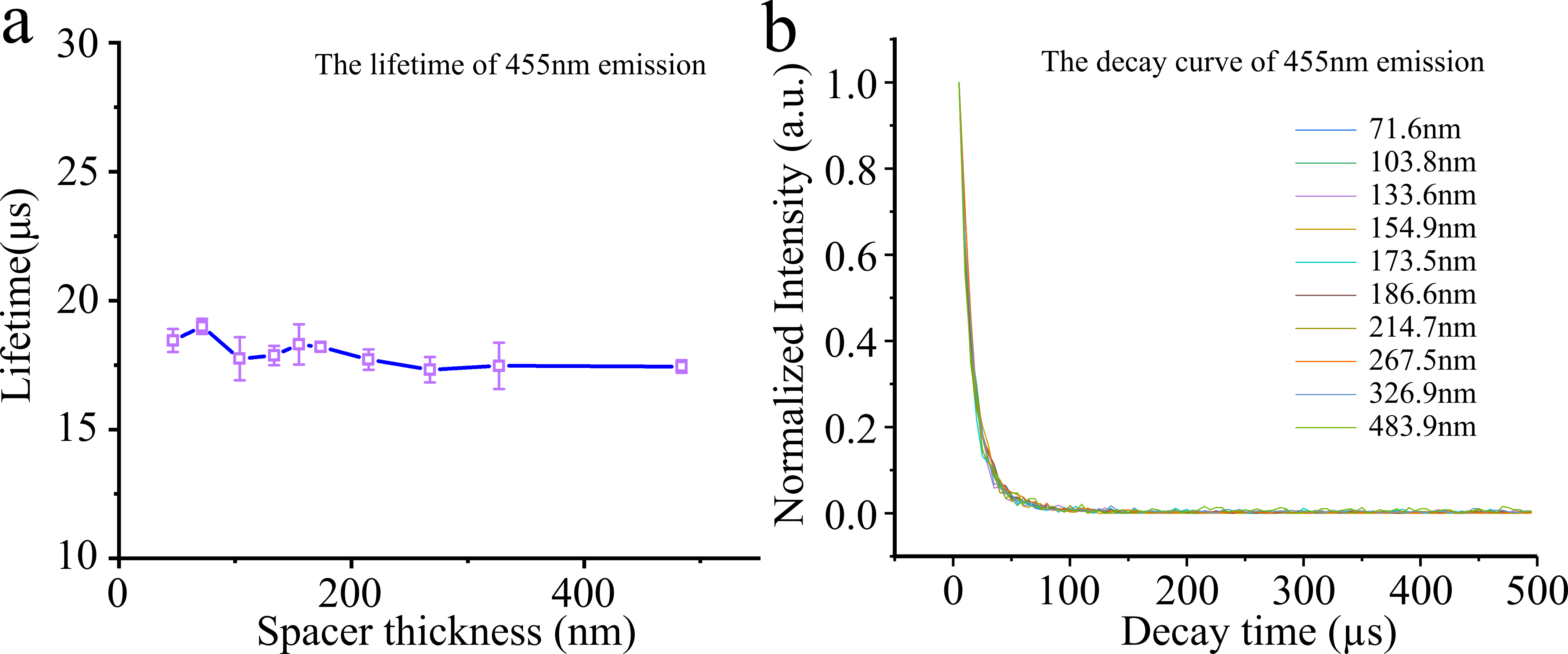


**Supplementary Figure 7.** Fluorescence lifetime of UCNPs at 455 nm**.** (a) The measured 455 nm fluorescence lifetime of UCNPs for different spacings between the particle’s centre and the mirror’s surface. Error bars are based on the standard deviation. (b) The measured 455 nm fluorescence decay curves

We now analyse the Purcell effect induced change on the emission rate and quantum yield. In the upconverting system, under strong excitation limit, without considering the cross-relaxation effect, the emission rate and the quantum yield will not be affected by the Purcell effect^11^, as the branching ratio is independent of the Purcell effect. Then the Purcell effect modulated equations are:

$\frac{dn_{1}}{dt}={-c}_{1}n_{1}n_{7}+{P_{u21}w}_{2}n_{2}+b_{31}\left( b_{31}P_{u31}+{b_{32}P}_{u32} \right)w_{3}n_{3}+b_{41}\left( b_{41}P_{u41}+{b_{42}P}_{u42}+b_{43}P_{u43} \right)w_{4}n_{4}+b_{51}\left( b_{51}P_{u51}+{b_{52}P}_{u52}+{b_{53}P}_{u53}+{b_{54}P}_{u54} \right)w_{5}n_{5}-k_{41}n_{1}n_{4}-k_{31}n_{1}n_{3}-k_{51}n_{1}n_{3}$ $\left( 10 \right)$

$\frac{dn_{2}}{dt}=c_{1}n_{1}n_{7}-c_{2}n_{2}n_{7}-b_{21}P_{u21}{w_{2}n}_{2}+b_{32}\left( b_{31}P_{u31}+{b_{32}P}_{u32} \right){w_{3}n}_{3}+b_{42}{\left( b_{41}P_{u41}+{b_{42}P}_{u42}+b_{43}P_{u43} \right)w}_{4}n_{4}+b_{52}\left( b_{51}P_{u51}+{b_{52}P}_{u52}+{b_{53}P}_{u53}+{b_{54}P}_{u54} \right){w_{5}n}_{5}+k_{41}n_{1}n_{4}+2k_{31}n_{1}n_{3}$ $\left( 11 \right)$

$\frac{dn_{3}}{dt}=c_{2}n_{2}n_{7}{-c}_{3}n_{3}n_{7}-{(b_{31}P_{u31}+{b_{32}P}_{u32})w}_{3}n_{3}+b_{43}{{(b_{41}P_{u41}+{b_{42}P}_{u42}+b_{43}P_{u43})w}_{4}n}_{4}+b_{53}{{(b_{51}P_{u51}+{b_{52}P}_{u52}+{b_{53}P}_{u53}+{b_{54}P}_{u54})w}_{5}n}_{5}+{2k}_{51}n_{5}n_{1}+k_{41}n_{4}n_{1}-k_{31}n_{3}n_{1}$ $\left( 12 \right)$

$\frac{dn_{4}}{dt}=c_{3}n_{3}n_{7}-c_{4}n_{4}n_{7}-{{(b_{41}P_{u41}+{b_{42}P}_{u42}+b_{43}P_{u43})w}_{4}n}_{4}+b_{54}{{(b_{51}P_{u51}+{b_{52}P}_{u52}+{b_{53}P}_{u53}+{b_{54}P}_{u54})w}_{5}n}_{5}-k_{41}n_{1}n_{4}$ $\left( 13 \right)$

$\frac{dn_{5}}{dt}=c_{4}n_{4}n_{7}-{(b_{51}P_{u51}+{b_{52}P}_{u52}+{b_{53}P}_{u53}+{b_{54}P}_{u54})w_{5}n}_{5}-k_{51}n_{1}n_{5}$ $\left( 14 \right)$

$\frac{dn_{7}}{dt}=\frac{P\delta}{h\nu}n_{6}-{P_{u76}w}_{7}n_{7}-\left( c_{1}n_{1}+c_{2}n_{2}+c_{3}n_{3}+c_{4}n_{4} \right)n_{7}$ $\left( 15 \right)$

Here $P_{ujk}$ (e.g. $P_{u31}, j=3, k=1$) is the Purcell factor for dipoles which have resonance frequency matches with the energy gap between level *j* and *k*. The $P_{ujk}$ is either for the dipoles parallel to the mirror surface (horizontal dipoles) or perpendicular to the surface (vertical dipoles), for the case of calculating the enhancement for *x/y* dipoles and *z* dipoles, respectively. We calculate the Purcell factors according to Chen et al.^12^ As an example, Supplementary Figure 8 shows the calculated $P_{u52}$ (related to 455 nm emission, Supplementary Figure 8a) and $P_{u31}$ (related 800 nm emission, Supplementary Figure 8b) for different spacing between the centre of the particle and the mirror surface.


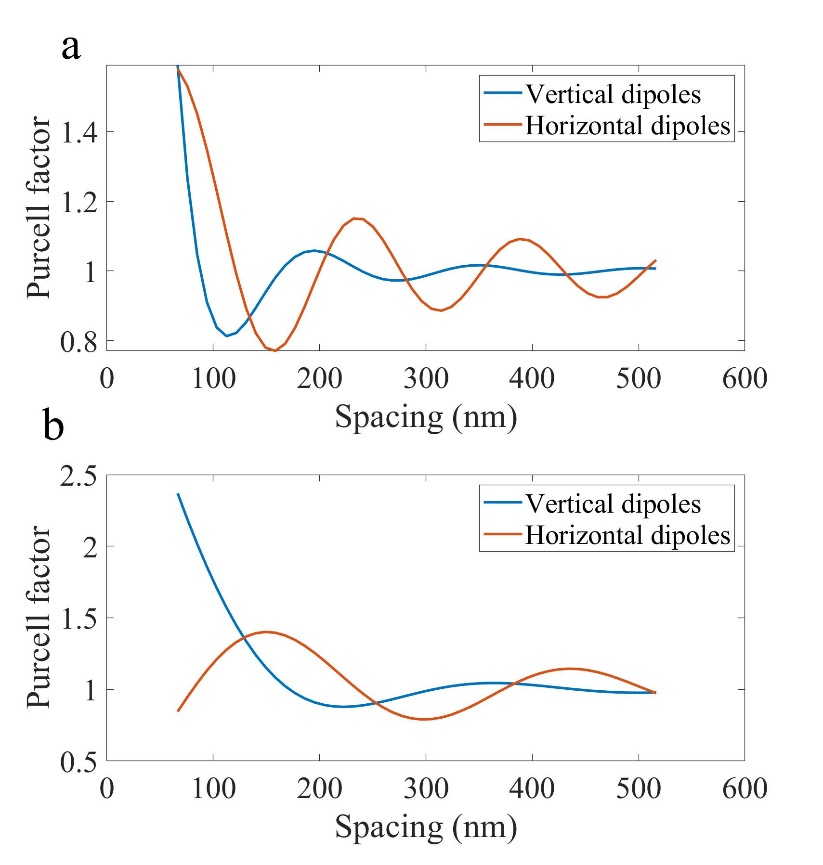


**Supplementary Figure 8.** Purcell factors from vertical and horizontal dipoles. Computed Purcell factor as a function of spacing (or distance of UCNP to mirror) with (a) 455 nm and (b) 800 nm emission wavelength for emitters oriented parallel or orthogonal to the mirrors’ surface.

From the plasmon modified equations, the change in decay rate from the Purcell effect will modify the steady-state carrier distribution, which will affect the emission intensity and the quantum yield. Based on the rate equation, the 455 nm emission photon number for unit time is $b_{52}{(b_{51}P_{u51}+{b_{52}P}_{u52}+{b_{53}P}_{u53}+{b_{54}P}_{u54})w_{5}n}_{5}$. Here $n_{5}$ is the carrier concentration at the energy level ^1^D_2_. The emission quantum yield for emission 455 nm is $b_{52}(b_{51}P_{u51}+{b_{52}P}_{u52}+{b_{53}P}_{u53}+{b_{54}P}_{u54})w_{5}/((b_{51}P_{u51}+{b_{52}P}_{u52}+{b_{53}P}_{u53}+{b_{54}P}_{u54})w_{5}+k_{51}\times n_{1})$. Supplementary Figure 9 and 10 show the 455 nm enhancement processes for vertical and horizontal orientated dipoles, respectively. For each of spacing value, there is one suit of steady-state distribution at each level. Supplementary Figure 9a and 10a are the enhancement ratio for the emission, ratio=1 stands for the emission is the same as that with no enhancement. This factor depends on both Purcell factors and the steady-state concentration $n_{5}$ (Supplementary Figure 9b and 10b). This emission enhancement ratio for different spacings is supposed to modify the weighting of the dipoles’ amplitudes during our PSF simulation. Here this enhancement ratio is negligible. Supplementary Figure 9c and 10c are the enhancement ratio for the emission (455 nm) quantum yield for vertical and horizontal orientated dipoles, respectively. This ratio depends on both Purcell factors and the steady-state concentration $n_{1}$ (Supplementary Figure 9d and 10d).


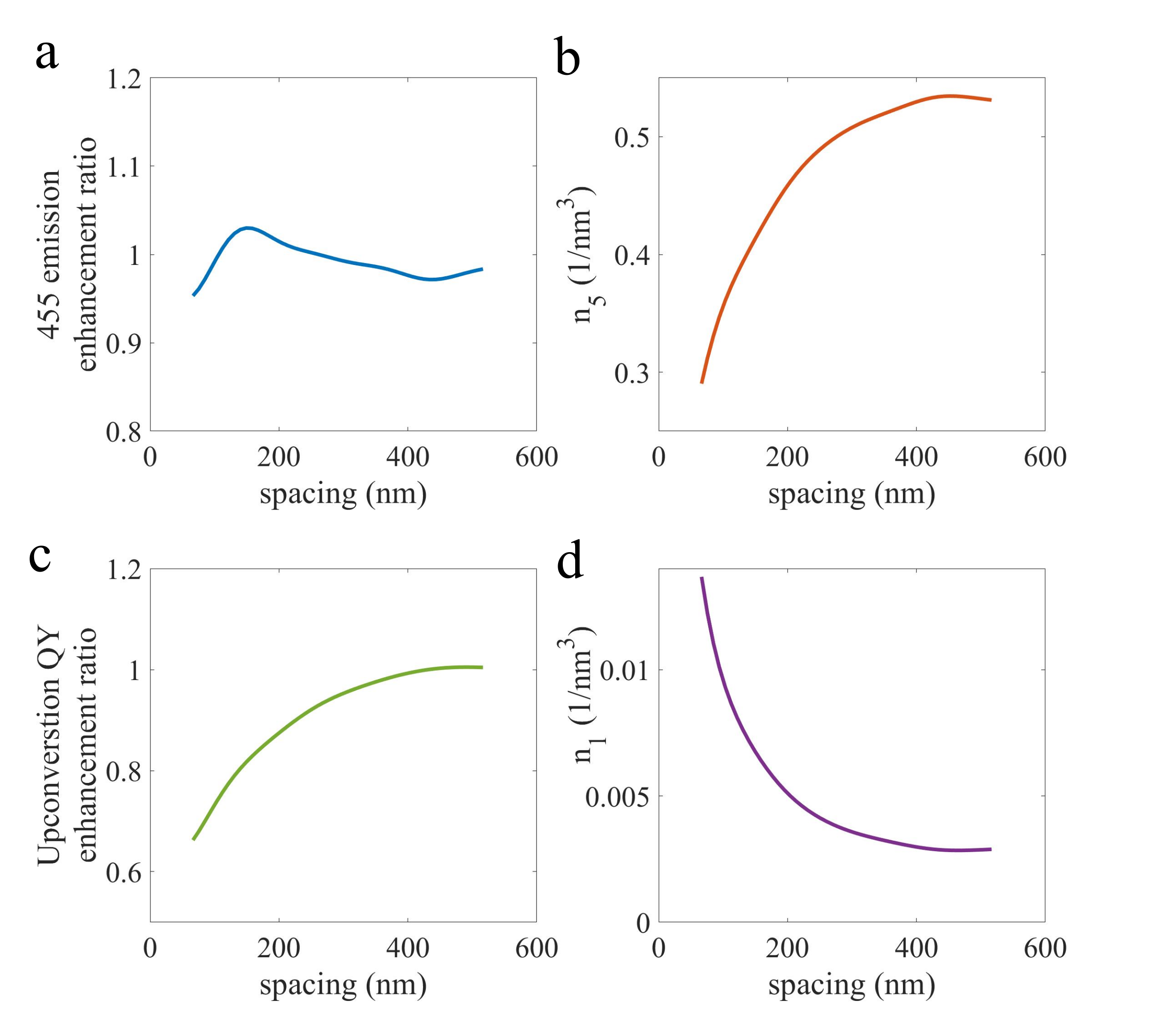


**Supplementary Figure 9.**  Enhancements on quantum yield and emission for vertically orientated dipoles. (a) Enhancement ratio for the 455 nm emission. (b) The carrier distribution at the energy level of $n_{5}$. (c) Enhancement ratio for the 455 nm emission quantum yield. (d) The carrier distribution at the energy level of $n_{1}$.


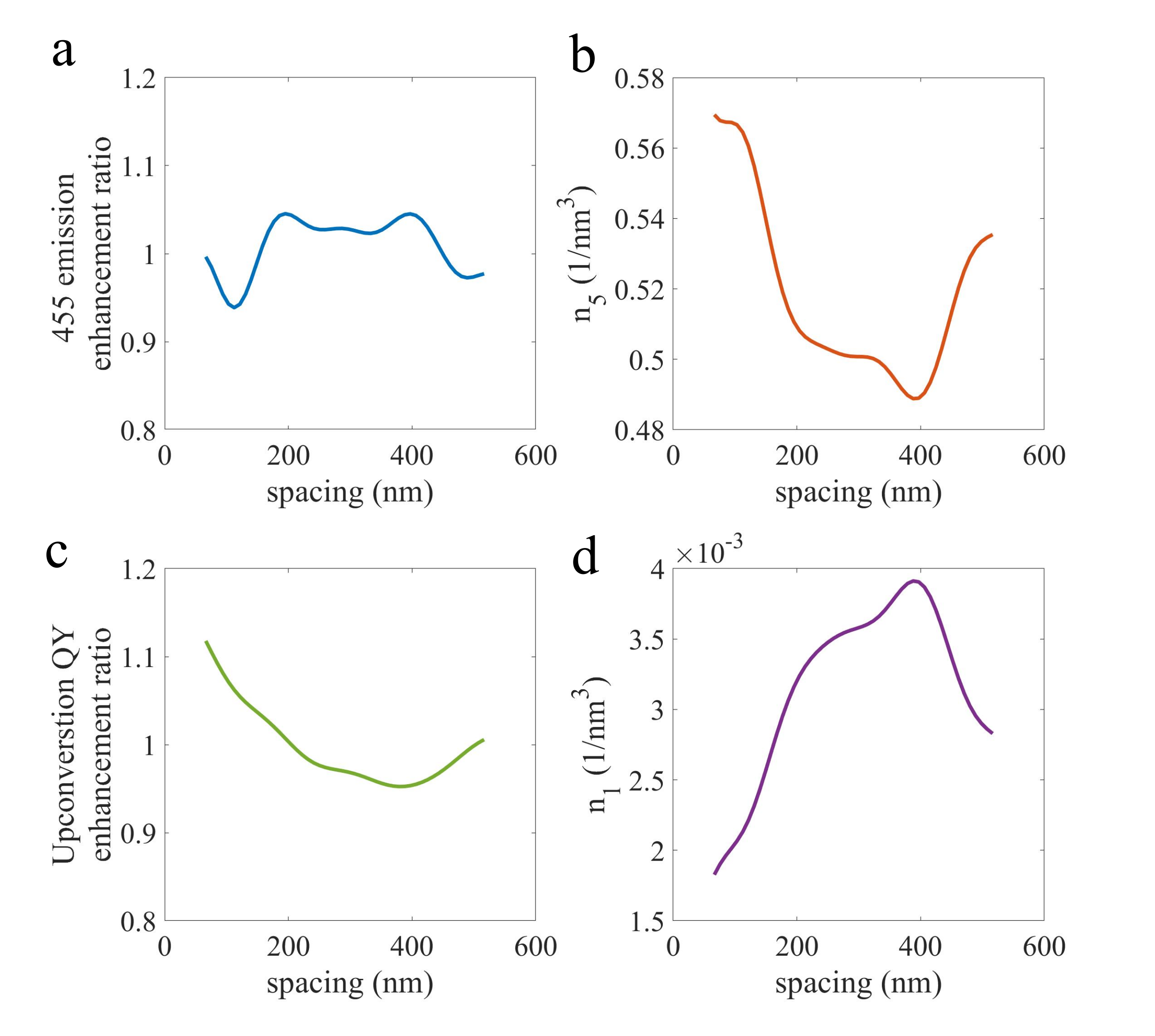


**Supplementary Figure 10.**  Enhancements on quantum yield and emission for horizontally orientated dipoles. (a) Enhancement ratio for the 455 nm emission. (b) The carrier distribution at the energy level of $n_{5}$. (c) Enhancement ratio for the 455 nm emission quantum yield. (d) The carrier distribution at the energy level of $n_{1}$.

1. *Comparison of simulated and experimentally observed PSFs for self-interference of UCNPs on mirror samples*

To simulate the self-interference effect of the UCNPs on the mirror, we calculated the interference pattern of the 455 nm emission band and the 800 nm emission band for different spacings (Supplementary Figure 11 and 12). Supplementary Figure 11 shows the complete simulation and experimental results of 455 nm emission. Supplementary Figure 11A-J shows the PSFs of 455 nm emission in the x-y plane with the spacing equal to 71.6 nm, 103.8 nm, 133.6 nm, 154.9 nm, 173.5 nm, 186.6 nm, 214.7 nm, 267.5 nm, 326.9 nm, and 483.9 nm, respectively. Figure 11K-T shows the PSFs in the *y-z* plane. Supplementary Figure 11a-j provides the experimental data in the *x-y* plane, which was captured by an EMCCD camera in the focus plane. Supplementary Figure 11k-t shows the measured self-interference pattern in the *y-z* plane. The experimental results nicely match with the simulation. Supplementary Figure 12 shows the corresponding simulation and experimental data at an emission wavelength of 800 nm.


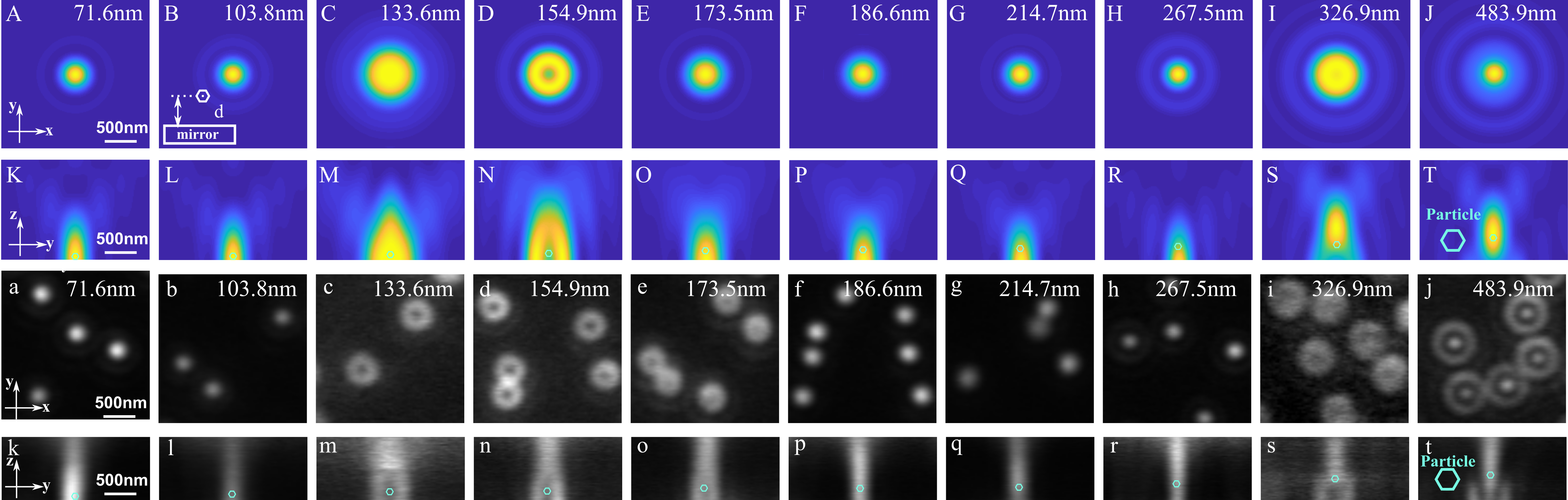


**Supplementary Figure 11.** Simulated and experimental measured distance-dependent 455nm emission PSFs of UCNPs with self-interference. The UCNP-to-mirror distance *d* (see inset in A) increases from the left to the right column as indicated from 71.6 nm to 483.9 nm. (A-T) The simulated PSFs of the self-interference for a particle on a mirror substrate with spacing distances of 71.6 nm, 103.8 nm, 133.6 nm, 154.9 nm, 173.5 nm, 186.6 nm, 214.7 nm, 267.5 nm, 326.9 nm, and 483.9 nm, at the *x-y* plane (A-J) and *y-z* plane (K-T). (a-t) The experimentally measured PSFs of the upconversion emission self-interference for UCNPs on a mirror substrate with the distance of 71.6 nm, 103.8 nm, 133.6 nm, 154.9 nm, 173.5 nm, 186.6 nm, 214.7 nm, 267.5 nm, 326.9 nm, and 483.9 nm away from the mirror surface, at the *x-y* plane (a-j) and *y-z* plane (k-t). The radius of the UCNPs was 16.5 nm, and the emission wavelength used for detection is at 455 nm. Scale bar is 500 nm. The imaging focal plane in (A-J) and (a-j) is at *z*=0, where the UCNP is located. The tiny cyan hexagon indicates the *z-y* plane position of the UCNPs, where *z*=0 and *y*=0. The experiment results were captured by an EMCCD with an exposure time of 20 ms. The experimental PSFs at the *z-y* plane is the cross-section images of a 3D volume image generated by stacking 160 defocused images (20 nm for step size) at the *x-y* plane. The defocussing is well-defined and achieve by the objective’s piezoelectric positioner.

**
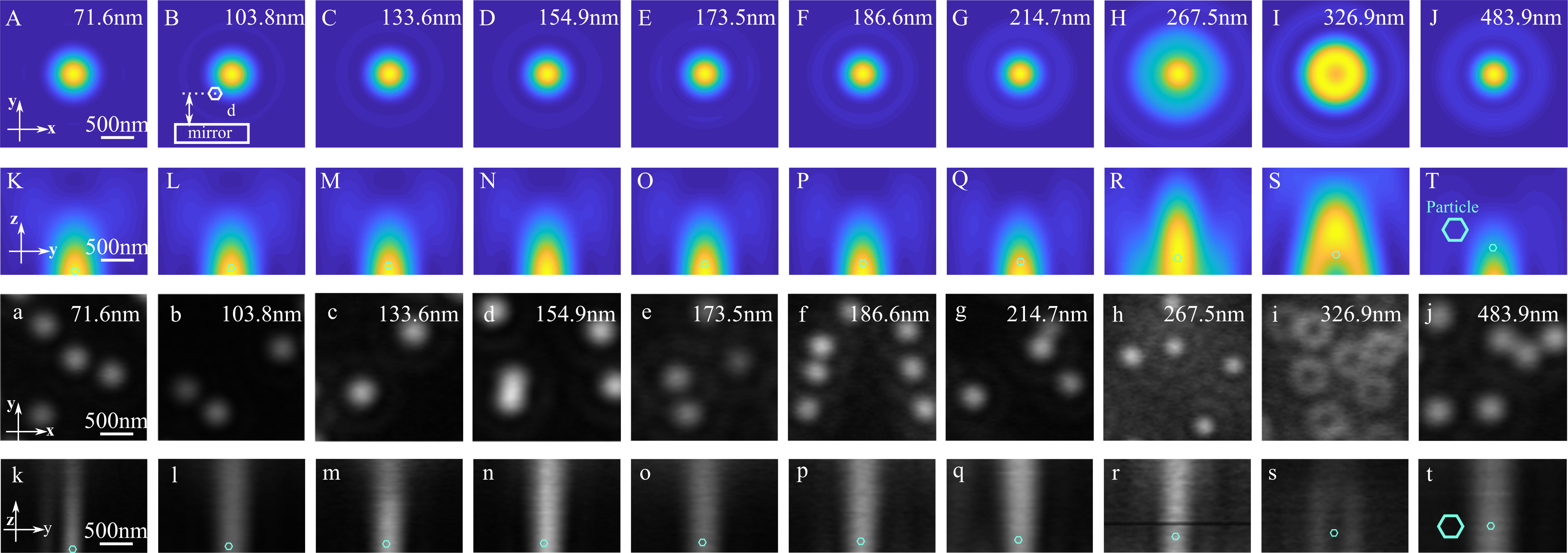
**

**Supplementary Figure 12.** Simulated and experimental measured distance-dependent 800 nm emission PSFs of UCNPs with self-interference. The UCNP-to-mirror distance *d* (see inset in A) increases from the left to the right column as indicated from 71.6 nm to 483.9 nm. (A-T) The simulated PSFs of the self-interference for a particle on a mirror substrate with spacing distances of 71.6 nm, 103.8 nm, 133.6 nm, 154.9 nm, 173.5 nm, 186.6 nm, 214.7 nm, 267.5 nm, 326.9 nm, and 483.9 nm, at the *x-y* plane (A-J) and *y*-*z* plane (K-T). (a-t) The experimentally measured PSFs of the upconversion emission self-interference for UCNPs on a mirror substrate with the distance of 71.6 nm, 103.8 nm, 133.6 nm, 154.9 nm, 173.5 nm, 186.6 nm, 214.7 nm, 267.5 nm, 326.9 nm, and 483.9 nm away from the mirror surface, at the *x-y* plane (a-j) and *y-z* plane (k-t). The radius of the UCNPs was 16.5 nm, and the emission wavelength used for detection is at 800 nm. Scale bar is 500 nm. The imaging focal plane in (A-J) and (a-j) is at *z*=0, where the UCNP is located. The tiny cyan hexagon indicates the *z-y* plane position of the UCNPs, where *z*=0 and *y*=0. The experiment results were captured by an EMCCD with an exposure time of 20 ms. The experimental PSFs at the *z-y* plane is the cross-section images of a 3D volume image generated by stacking 160 defocused images (20 nm for step size) at the *x-y* plane. The defocussing is well-defined and achieve by the objective’s piezoelectric positioner.


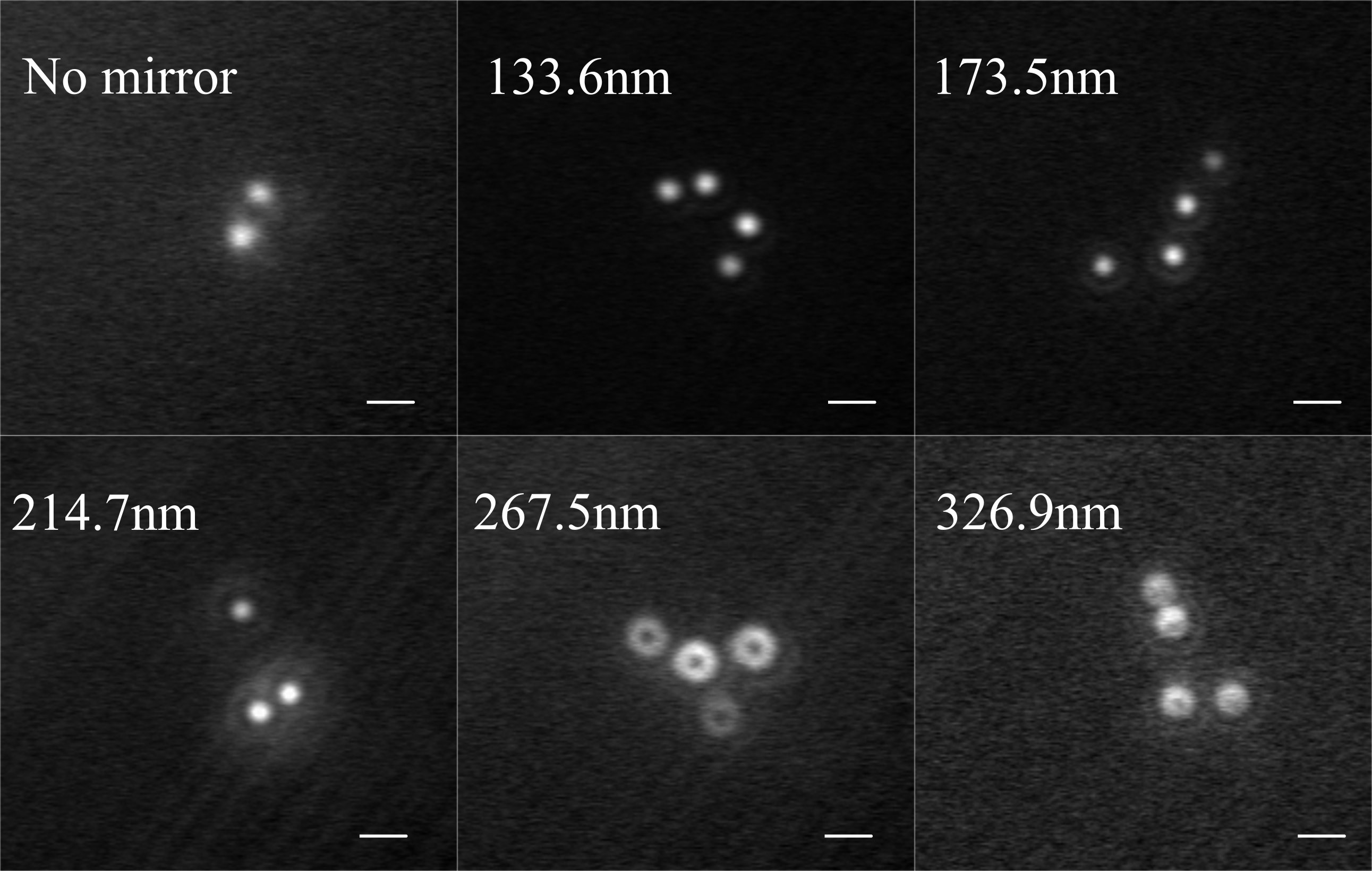


**Supplementary Figure 13**. Measured self-interference PSFs for 650 nm emission. The experimentally measured PSFs of the upconversion emission self-interference for UCNPs on a mirror substrate with the distance of 133.6 nm, 173.5 nm, 214.7 nm, 267.5 nm, and 326.9 nm away from the mirror surface, The radius of the UCNPs was 16.5 nm, and the emission wavelength used for detection is at 650 nm. Scale bars are 500 nm.


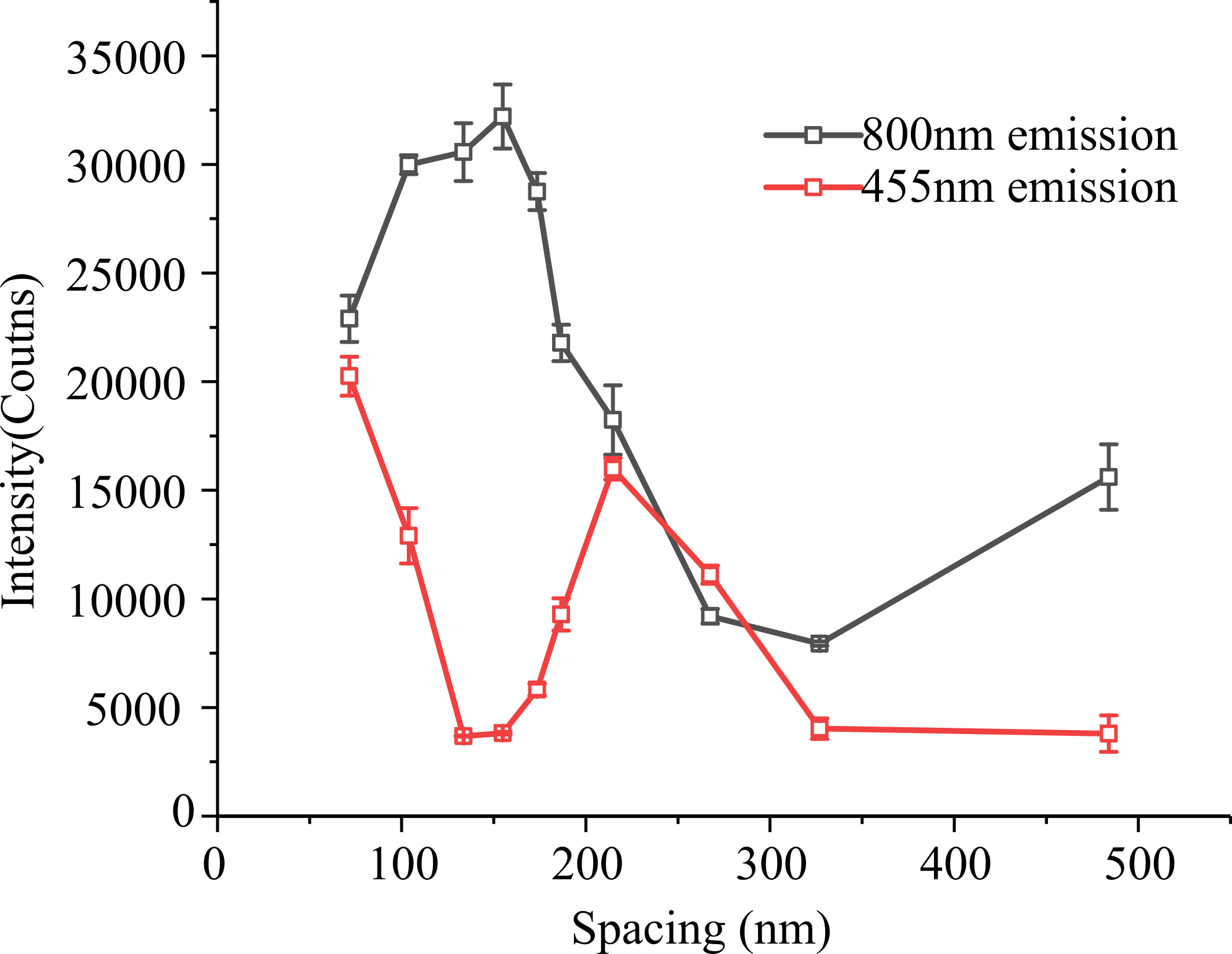


**Supplementary Figure 14**. Integrated collected emission intensity for different spacing values. Error bars are based on the standard deviation.

1. *Particle size effect to PSF*

In our system, the feature of light field distribution is much larger than the size of the nanoparticle; hence, the lateral distribution of emitters in 33 nm will not affect the diffraction-limited emission PSF. As a contrast, the axial distribution of emitters within a nanoparticle region will modify the PSF. At the Supplementary Figure 15, we simulated the self-interference effect by integrating 204 emitters randomly distributed over the volume of a 33 nm nanoparticle. Supplementary Figure 15 c and 15 d are the simulated *x-y* PSF for the spacing of 154.9nm with the emitter at the centre of particle and randomly distributed over the particle, respectively. The axially distributed emitters inside the particle will “blur” the PSF, as the PSF of the particle is a linear combination of individual PSFs from each emitter that have slightly different axial positions. Hence, the simulated calibration curve for a particle with emitters randomly located in its volume can be simplified as the convolution of a standard calibration curve (all emitter is at the centre point of the particle) with a top-hat function with a width of d (diameter of the particle). A larger size particle will decrease the gradient of the calibration curve and reduce the resolution. The PSFs for spacings of 71.6 nm (Supplementary Figure 15b) and 483.9 nm (Supplementary Figure 15f) have been less affected by the random emitters, as the modulation strength to PSF depends on the gradient of the characterization curve.


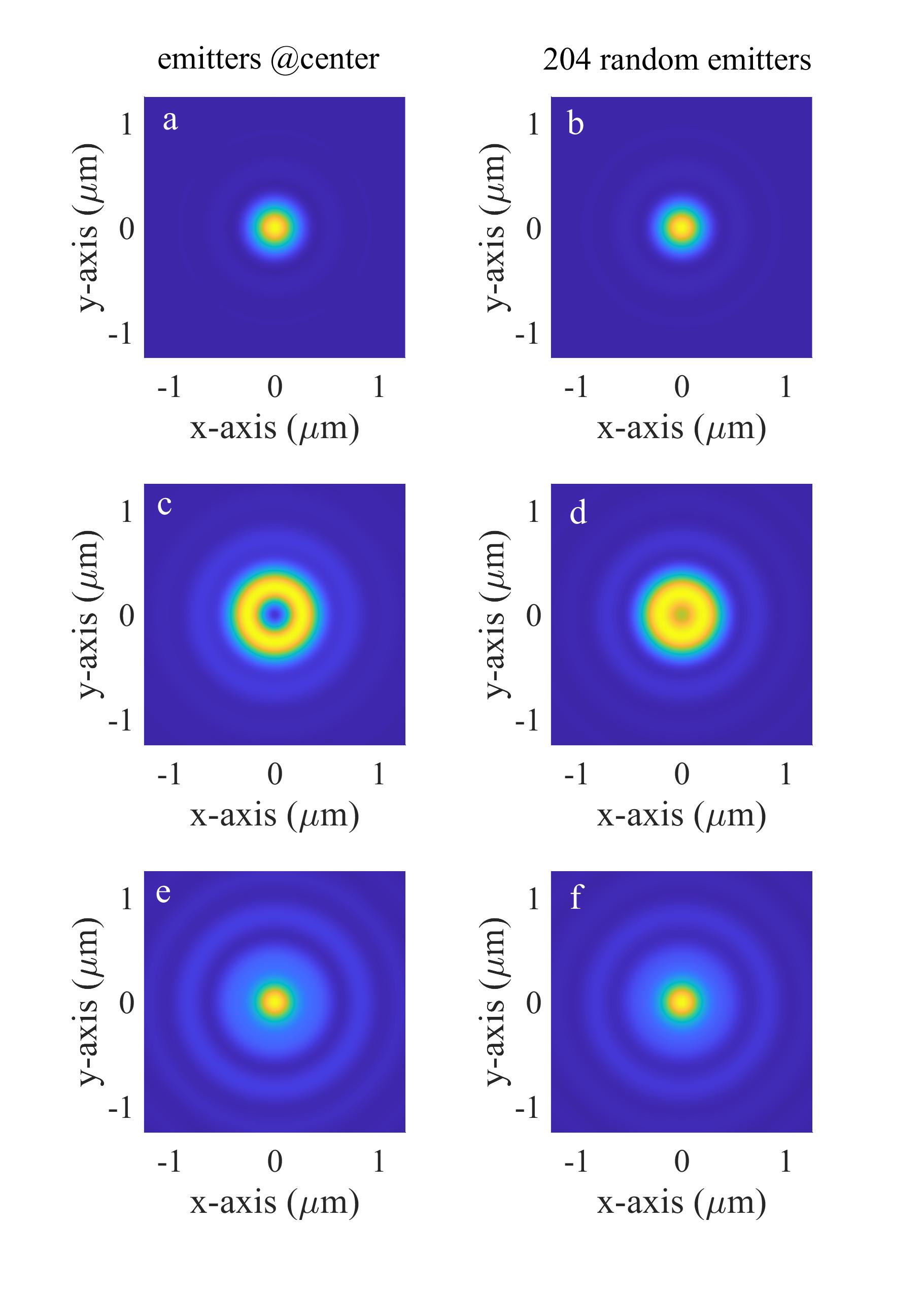


**Supplementary Figure 15.** The simulated PSFs with centralized and randomly distributed emitters. The simulated *x-y* PSFs for UCNPs with spacings of 71.6 nm (a, b), 154.9 nm (c, d) and 483.9 nm (e, f). Three emitters are located at the centre of a nanoparticle. (a, c, e). 204 emitters are located randomly inside the nanoparticle (b, d, f).


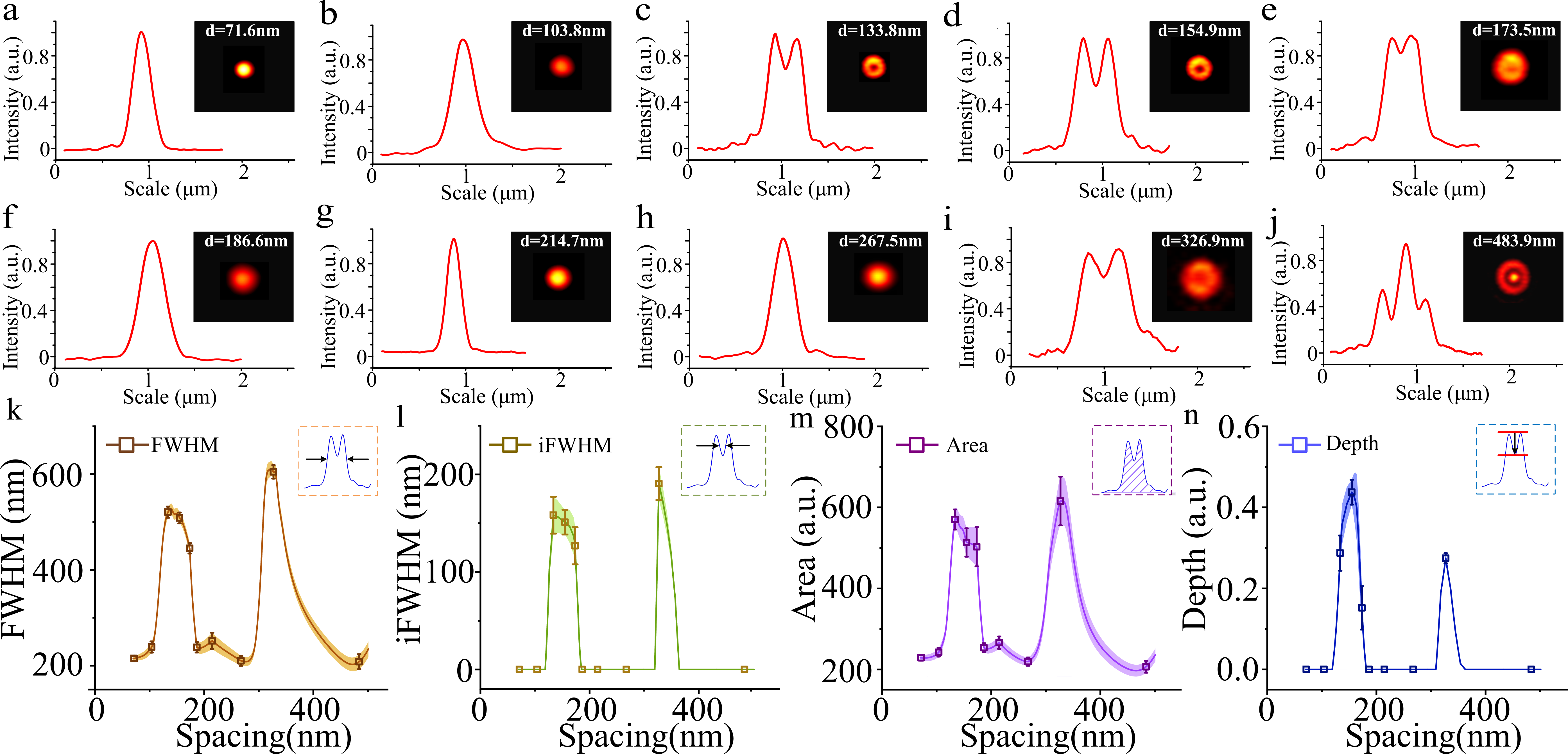


**Supplementary Figure 16**. Variation of feature from the observed 455 nm emission PSF used to determine the distance between UCNP and mirror. (a-j) Cross-section through the PSF of UCNPs (shown as insets) at spacing distances of 71.6 nm, 103.8 nm, 133.6 nm, 154.9 nm, 173.5 nm, 186.6 nm, 214.7 nm, 267.5 nm, 326.9 nm, and 483.9 nm. (k-n) The four characteristic parameters of the PSF, *FWHM*, *iFWHM*, *Area*, and *Depth*, respectively, measured as a function of the distance between UCNP and mirror. Error bars are based on the standard deviation.


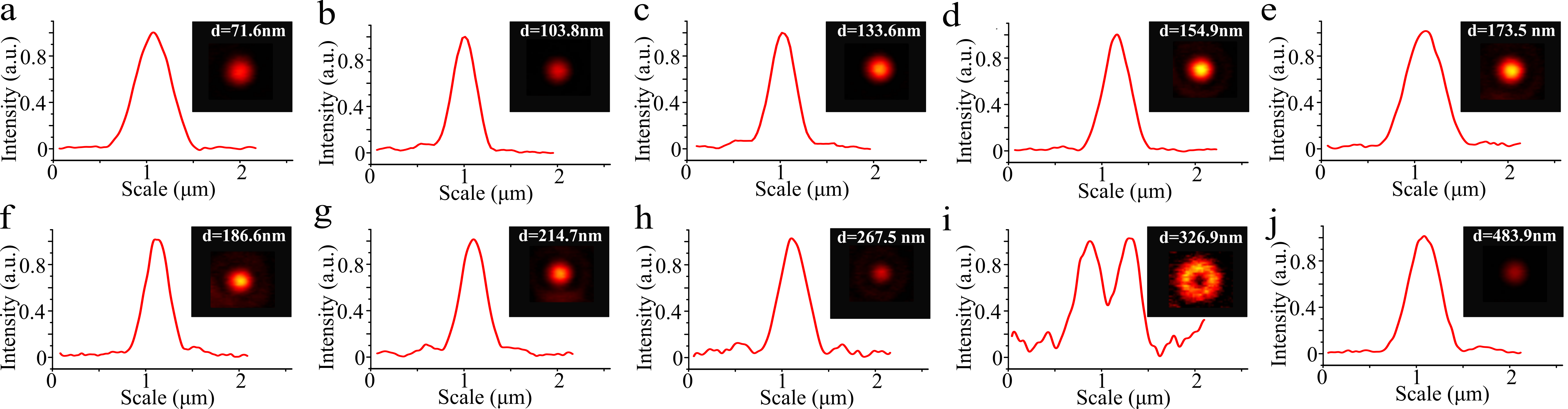


**Supplementary Figure 17**. Variation of feature from the observed 800 nm emission PSF used to determine the distance between UCNP and mirror. (a-j) Cross-section through the PSF of UCNPs (shown as insets) at spacing distances of 71.6 nm, 103.8 nm, 133.6 nm, 154.9 nm, 173.5 nm, 186.6 nm, 214.7 nm, 267.5 nm, 326.9 nm, and 483.9 nm.

**Supplementary Note 5: The sensing method and estimation of resolution**

As described in the main text, a measured PSF pattern will generate four parameters (*FWHM*, *iFWHM*, *Area*, and *Depth*). Comparing the parameters with the calibration curve (Figure 3g-j) enables the estimation of the corresponding spacing. Note that some of the parameter values will indicate several irrelevant spacing values (as the curve is not mono-linear), but the other parameters generated by these irrelevant values will not match with the measured other parameters. Hence, by analysing four parameters at the same time, a single corresponding spacing value will be found for a PSF pattern. The calibration error for four curves is shown in Supplementary Figure 18, where the thick coloured lines are the error region calculated by the measured error of the parameters. The sensing resolution is estimated from these error regions. Taking an *FWHM* value (429.6nm) from one UCNP from Step 2 (Figure 4) as an example, a known parameter value indicates a spacing value (121.1 nm) in the calibration curve (Supplementary Figure 18a). The accuracy of the spacing value is half the length of the cross-section of the error regions with the *FWHM* value (shown at the inset of Supplementary Figure 18a). These accuracy values are the sensing resolution for our method. It is worth to mention that in terms of the estimation of spacing and its resolution, both *iFWHM* and *Depth* are only useful when their value is non-zero.

If the characterizing value is within the error range of an inflexion point (e.g. 557 < Area < 676), the cross-section of the error region must go across both the sections with spacing larger and smaller than the inflexion point, as illustrated in Supplementary Figure 18c (inset).

To estimate the spacing within an unknown range, a cross-validation method that considering all four parameters is required. Supplementary Figure 19 shows an example of using the method. (1) The values of *FWHM*, *iFWHM*, *Area* and *Depth* can be directly measured from the 2D PSF (Supplementary Figure 19a, inset). We denote these four values as *a_0_, b_0_, c_0_, d_0_* here. (2) According to the measured *FWHM*, multiple corresponding spacing values (in this case denoted as *Z_a1_, Z_a2_, Z_a3_, Z_a4_*) will match according to the calibration curve, as shown in Supplementary Figure 19a. Similarly, according to the measured *iFWHM*, *Area* and *Depth* values, three more sets of spacing values (denoted as *Z_bi_, Z_ci_, Z_di_*, with *i*=1,2,3,4) will be found as shown in Supplementary Figure 19b, S19c and S19d, respectively. Altogether, we obtained *j* potential spacing values (e.g. we have *j*=16 here for four suits of data). (3) For each of potential distances, e.g. *Z_a1_*=130.2 nm four characterizing values (*a_a1_*=*a_0_, b_a1_, c_a1_, d_a1_*) are found from the calibration curves, as shown in Supplementary Figure 19a, 19b, 19c and 19d. (4) We use Var_*j*= $\sum_{k=a}^{k=d} {(k_{0}-k_{j})}^{2}$ to rank each of four potential spacing values (e.g. Var_*Za1*=$\sum_{k=a}^{k=d} {(k_{0}-k_{a1})}^{2}$). (5) The smallest Var_*j* then most likely heralds the real distance value.

The calculated resolution curve by using four calibration curves is shown in Supplementary Figure 20a. As the slopes for the different spacing regions at four curves are changing, the resolution is different for different spacing. Hence, to achieve better resolution, the final spacing value should be estimated from the calibration curves that have the best resolution for the spacing range. The selection rule is shown in Supplementary Figure 20b, where 1, 2, 3, and 4 indicate the selection of the curves of *FWHM*, *Area*, *iFWHM and* *Depth*, respectively. Supplementary Figure 20c shows the final resolution curve, where a sub-25nm resolution can be achieved from 75 nm to 447 nm. It is notable that at the spacing range of 104-137 nm, 160-186 nm and 282-365 nm, the method provides a sensing resolution better than 5 nm.

**
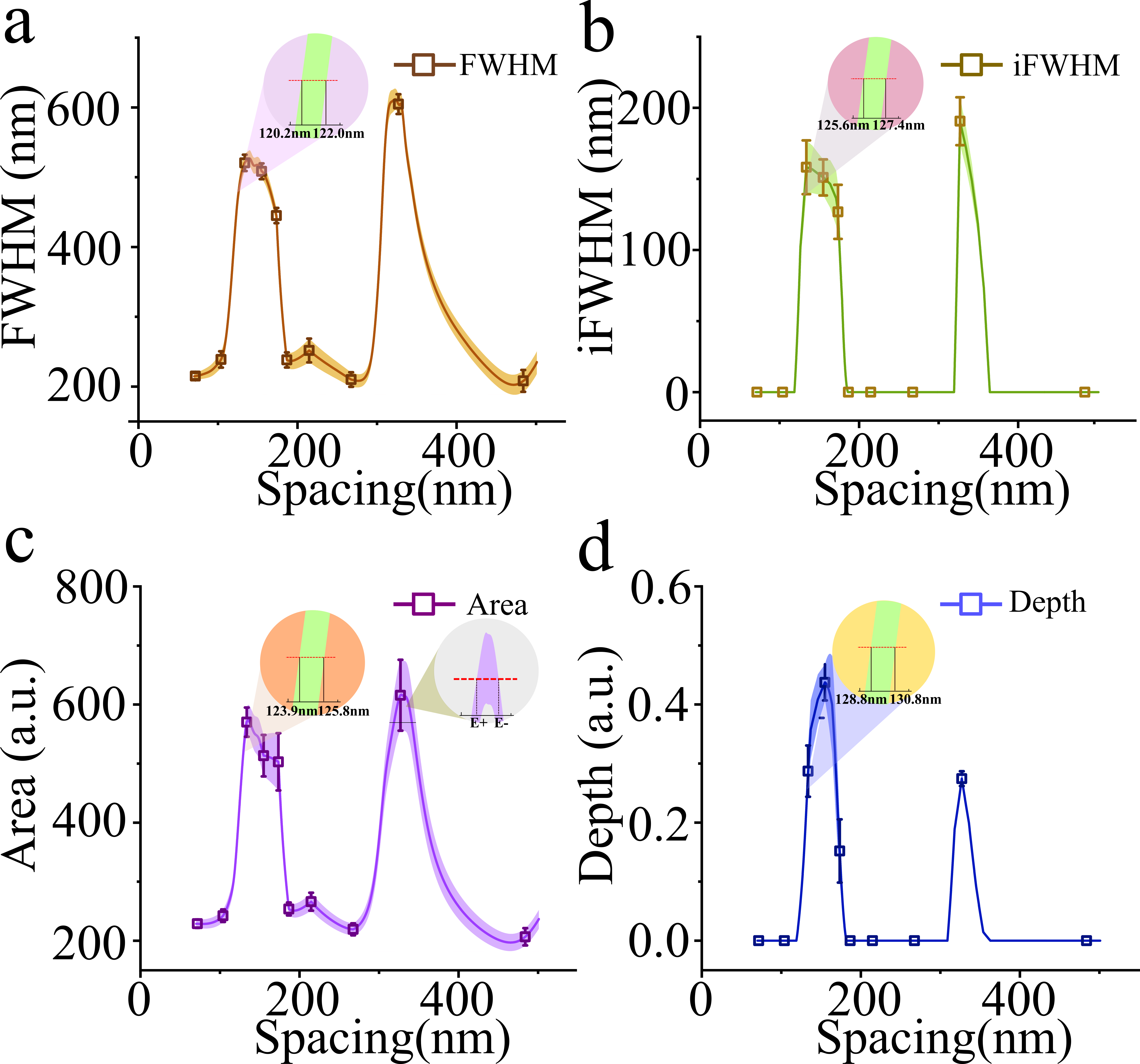
**

**Supplementary Figure 18.** The schematic of axial distance sensing method. The calibration error region of the (a) *FWHM*, (b) *iFWHM*, (c) *Area*, and (d) *Depth*. The sensing resolution is estimated, as shown in the inset. Error bars are based on the standard deviation.


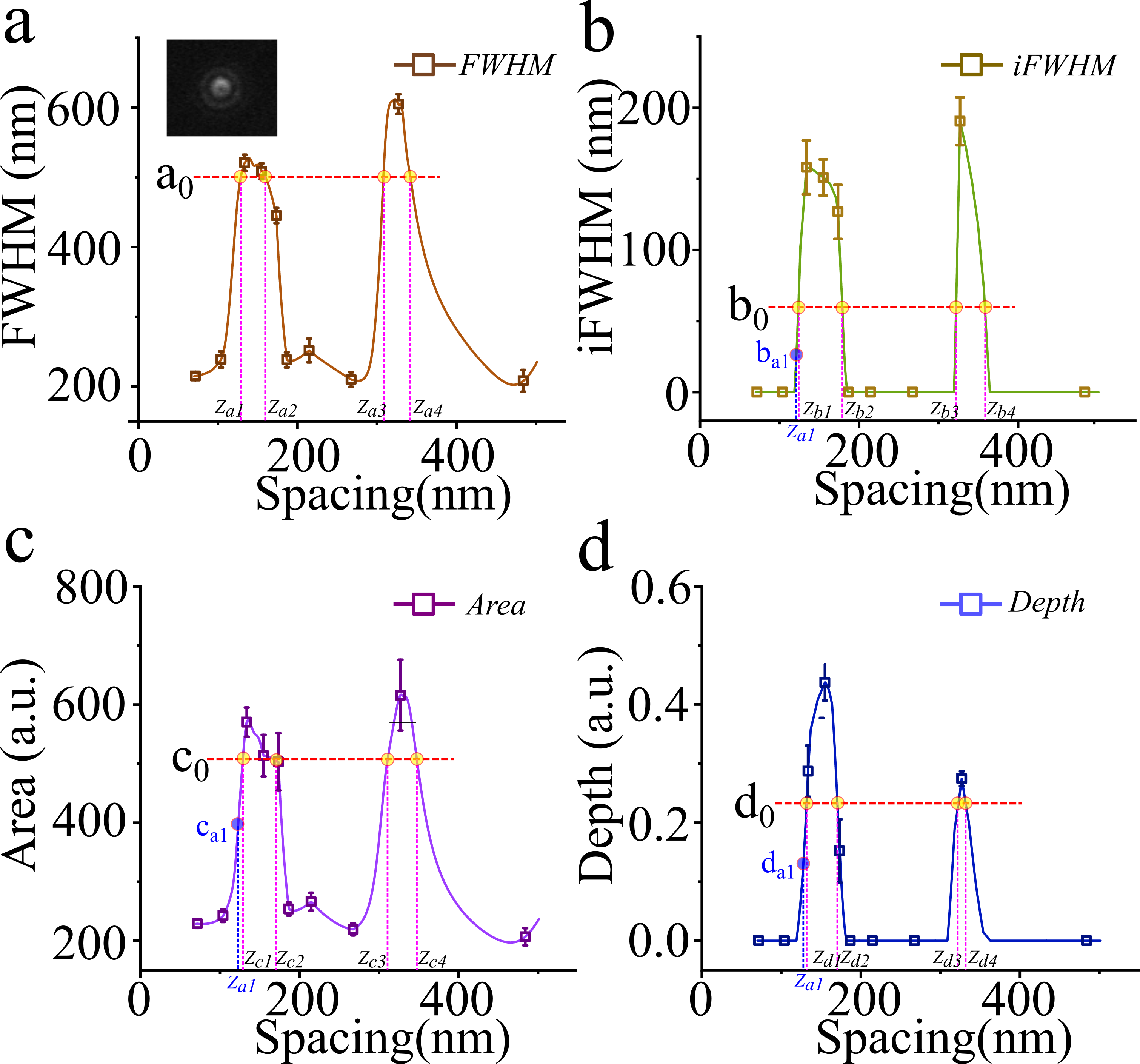


**Supplementary Figure 19**. Diagram of the cross-validation method to evaluate the distance from a PSF pattern. The characteristic parameters curves of the (a) *FWHM*, (b) *iFWHM*, (c) *Area*, and (d) *Depth*. The inset in (a) is a testing PSF pattern. Error bars are based on the standard deviation.


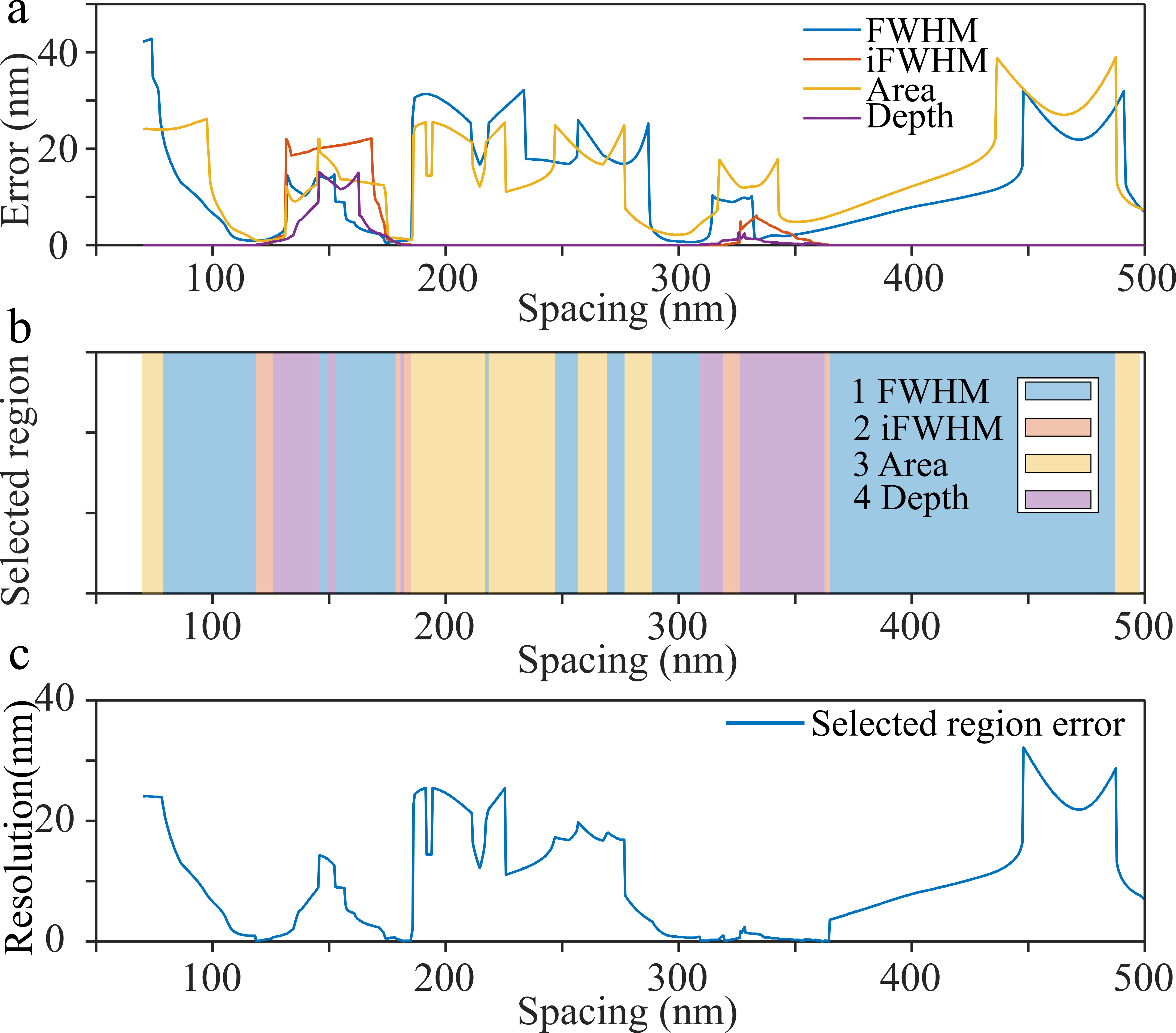


**Supplementary Figure 20.** Estimation of resolution by using the calibration curves for axial location sensing. (a) Axial locating errors for the localization precision of the derived spacing for four calibration curves (*FWHM*, *Area*, *iFWHM*, *Depth*). (b) The selection rule for obtaining the best resolution values at the different spacing regions. “1”, “2”, “3” and “4” indicate the selection of *FWHM*, *Area*, *iFWHM*, and *Depth*, respectively. (c) Resulting best values for the localization precision by using the self-interference effect.


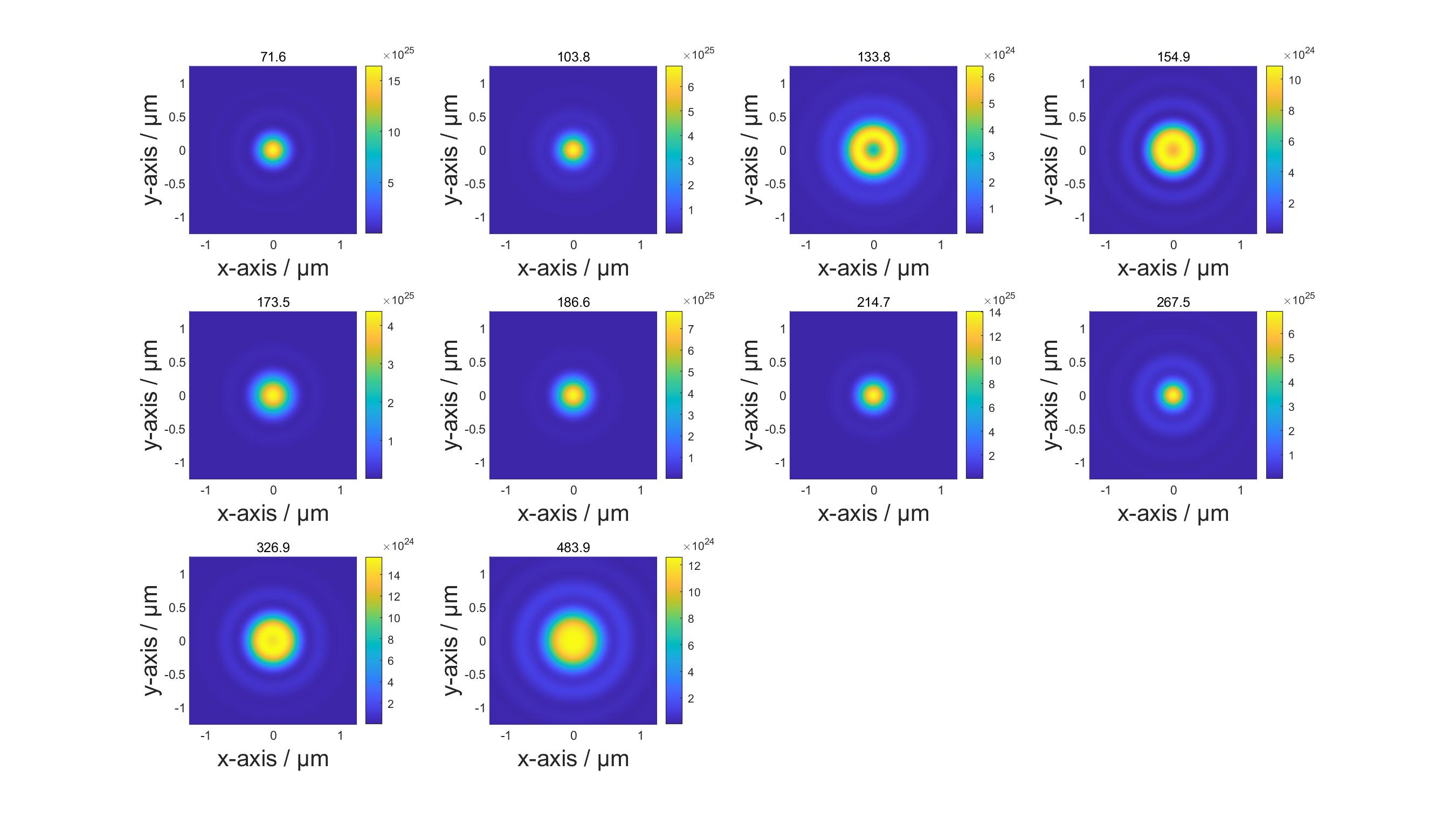


**Supplementary Figure 21.** Simulated self-interference PSF with water as the surrounding medium. The simulated PSFs of the self-interference for a particle on a mirror substrate with spacing distances of 71.6 nm, 103.8 nm, 133.6 nm, 154.9 nm, 173.5 nm, 186.6 nm, 214.7 nm, 267.5 nm, 326.9 nm, and 483.9 nm, at the *x-y* plane. The surrounding medium is water with a refractive index of 1.33.


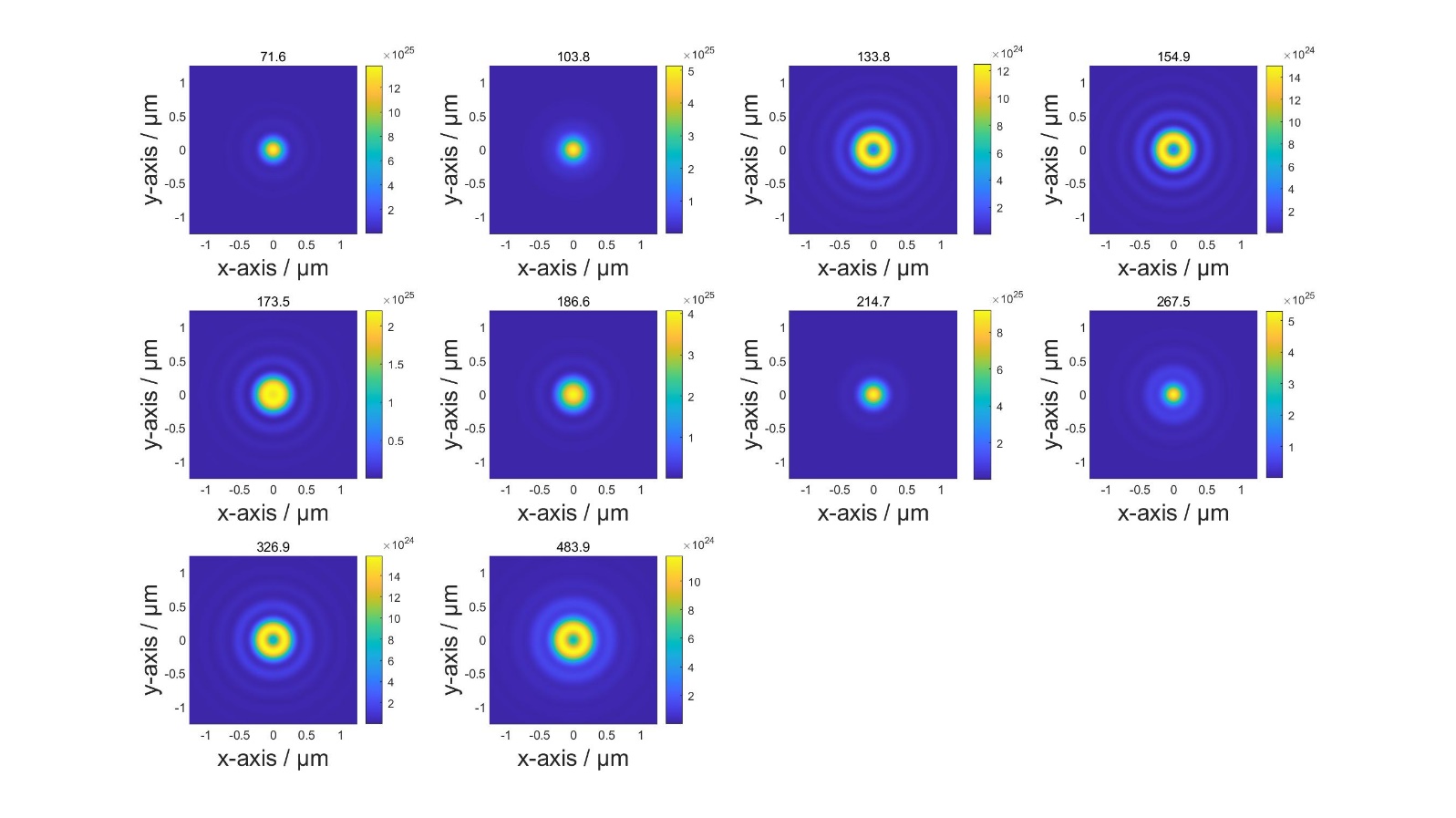
**Supplementary Figure 22.** Simulated self-interference PSF with air as the surrounding medium. The simulated PSFs of the self-interference for a particle on a mirror substrate with spacing distances of 71.6 nm, 103.8 nm, 133.6 nm, 154.9 nm, 173.5 nm, 186.6 nm, 214.7 nm, 267.5 nm, 326.9 nm, and 483.9 nm, at the *x-y* plane. The surrounding medium is air with a refractive index of 1.

**Supplementary Note 6: Estimation of the viscosity**

We calculate the viscosity according to the Stokes-Einstein equation:

$D=\frac{kT}{6\pi\eta R}$ (16)

where *D* is the diffusion coefficient, *k* is the Boltzmann constant, *T* is the temperature, *R* is the dynamic radius of the particle and *η* the viscosity of the medium. The example of the viscosity measuring method can be found in ref. ^13^.

The diffusion coefficient is calculated by fitting the mean-square displacement (MSD) into the below equation:

$\langle r^{2}\left( t \right)\rangle=2d\cdot D\cdot t^{\alpha}$ (17)

where *r²* is the MSD, r is the mean distance from the starting point that a particle diffuse in time *t*, *d* is the dimension (*d* = 2 for two-dimensional diffusion, *d* = 3 for three- dimensional diffusion), *D* is the diffusion coefficient, and *t* is the time step. The diffusive exponent *α* is a parameter differentiating anomalous from normal diffusion (i.e: When *α* < 1 the process is sub-diffusive and when *α*> 1 it is super-diffusive). In this work, to retrieve the value of *α*, the first 40% of the MSD points were plotted as log (*r²*) vs log (*t*) and fitted by a straight line. The slope of the MSD determines the diffusive exponent α. The MSD was calculated using the msdanalyzer MATLAB code that was written by Jean-Yves Tinevez^14^.


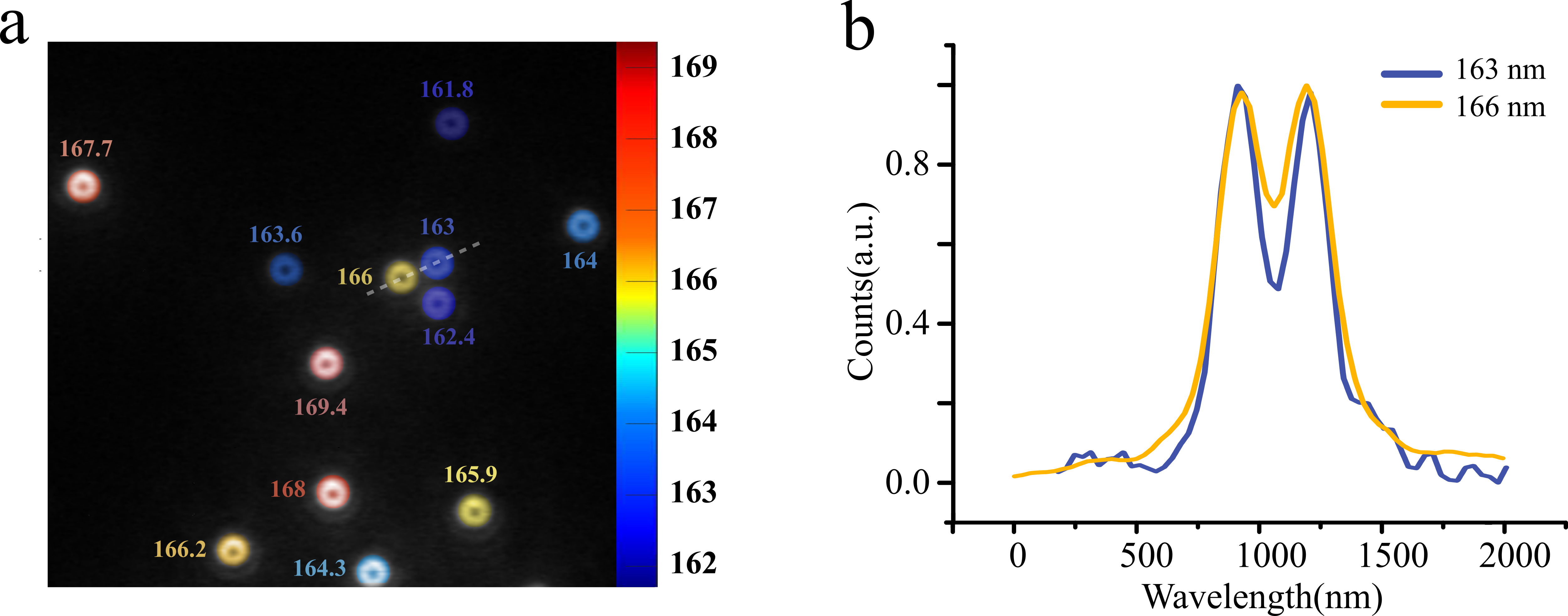


**Supplementary Figure 23.** Large field distance sensing of UCNPs. (a) The 2D fluorescence image of UCNPs on a mirror surface. The characterized thickness of the spacer is 145 nm. The UCNPs are from two batches of samples with averaged diameters of 32.8+/- 1.28 nm and 41.67+/- 1.54 nm. The averaged centre height for particle batch 1 and 2 are 163.2 +/- 1 nm and 167.2 +/- 1.4 nm respectively. (b) The cross-section of two typical UCNPs in (a). The measured distances from the particles’ centre to mirror surface are 166 nm and 163 nm. The SEM images and the size distribution of particles are shown in Supplementary Figure 25.


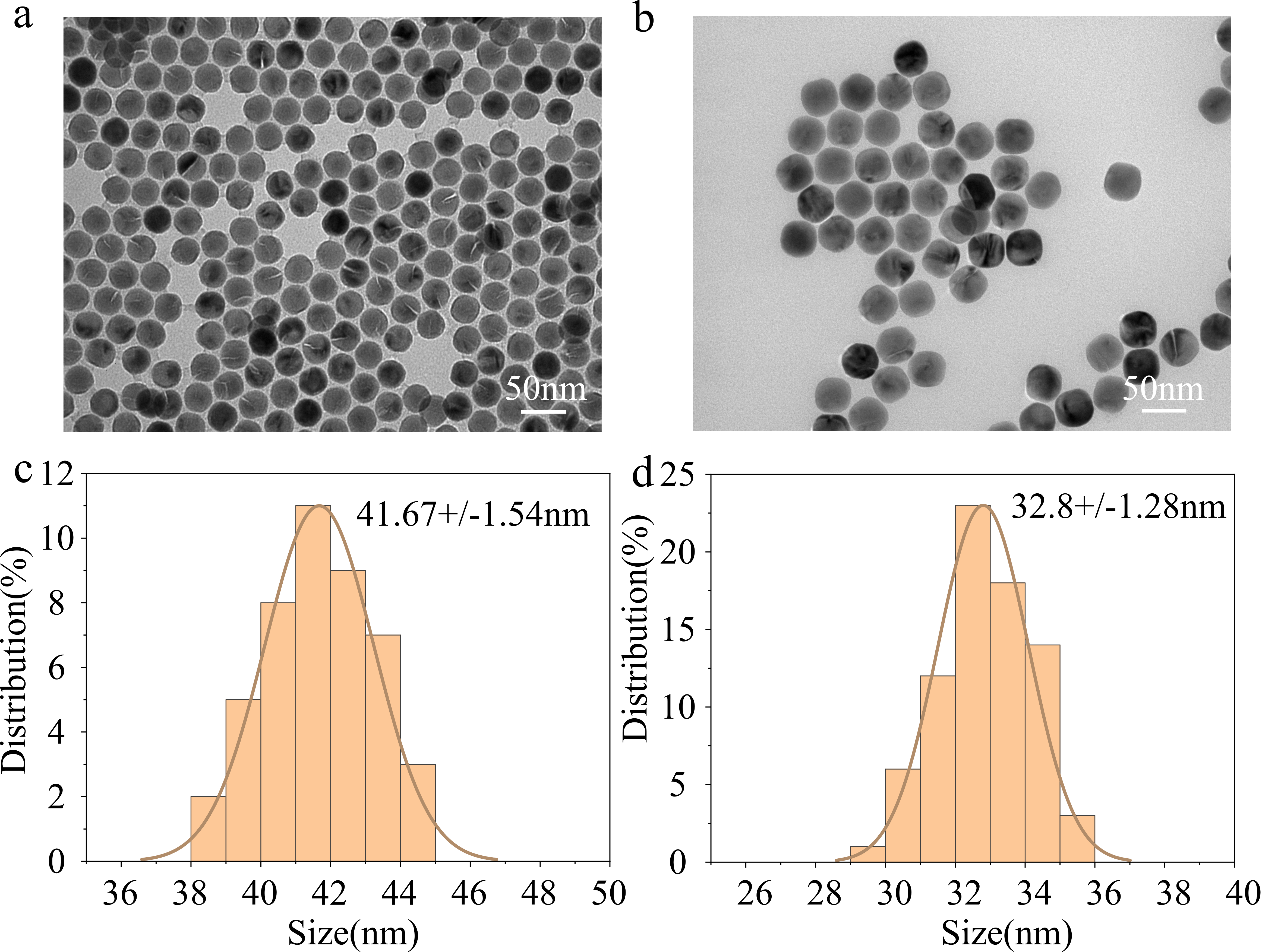


**Supplementary Figure 24.** TEM images (Top) and size distribution histograms (Bottom) of the nanoparticles. a) NaYF_4_: 20% Yb, 4% Tm. (b) NaYF_4_: 20% Yb, 4% Tm. (c) The size distribution of nanoparticles in (a). (d) The size distribution of nanoparticles in (b). Scale bar is 50 nm.

**Supplementary Note 7: Eliminating the error of feature values from the out-of-focus PSFs.**

Supplementary Figure 25a shows the self-interference pattern at different out of focus planes. The spacing is 154.9 nm. The focus is set at the axial position of the UCNP (154.9 nm from the surface), and the defocussing ranges are -100 nm to 120 nm away from the focus plane. The characteristic feature values *FWHM*, *iFWHM*, *Depth*, and *Area*, were derived from each self-interference pattern at different out-of-focus planes. The variation of these four parameters with defocussing is quite small, as shown in Supplementary Figure 25b. Then the relative variation of each parameter is shown in Supplementary Figure 25c. Defocusing of +/- 100 nm will only induce relative variations less than 2.3%, 3.3%,1.2% and 4.4% for *FWHM*, *iFWHM*, *Area*, and *Depth*, respectively.


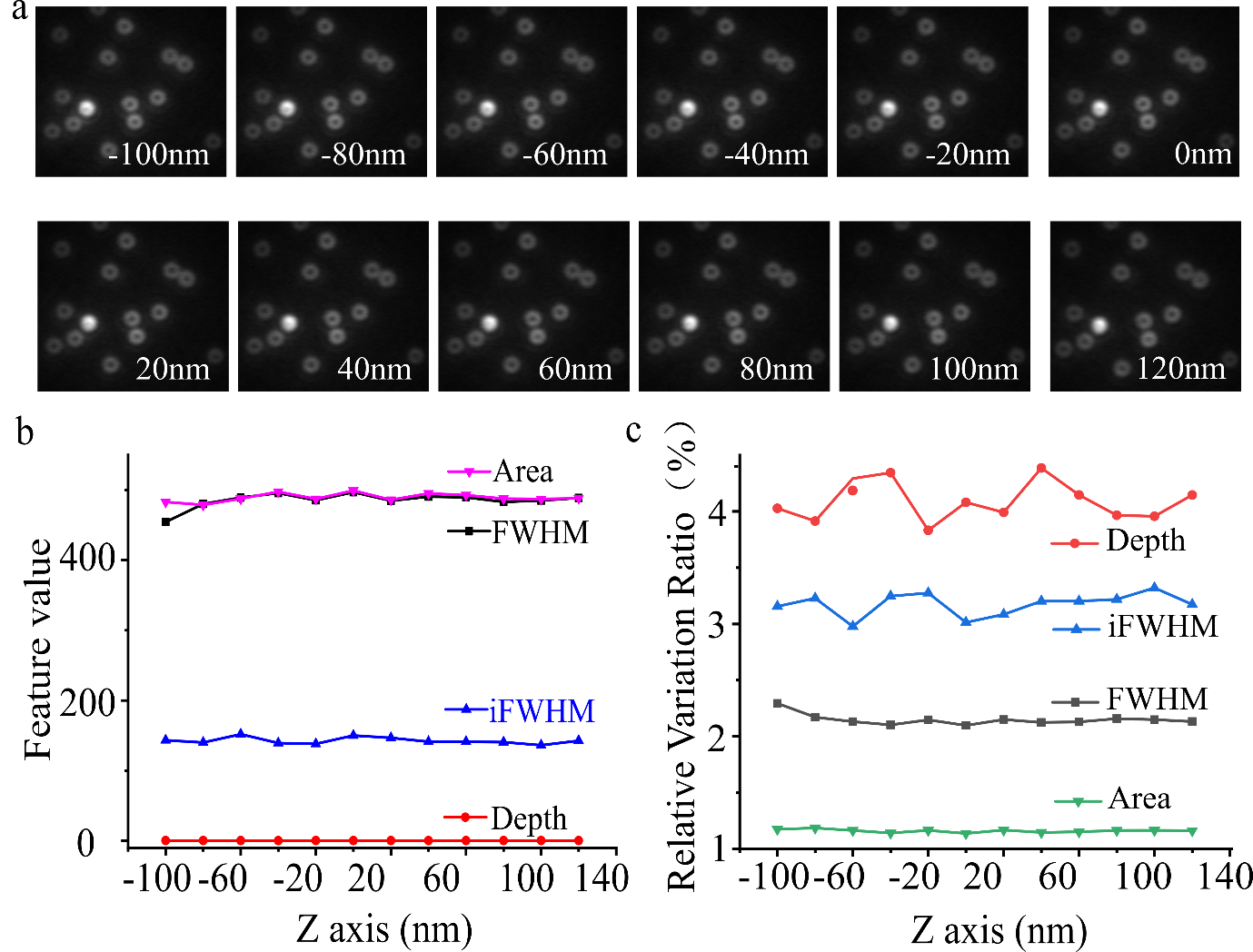


**Supplementary Figure 25.** The error of feature values from the out-of-focus PSFs**.** (a) The self-interference pattern at different out-of-focus planes. The focus plane corresponds to *z_defoc_*= 0nm. (b) The characteristic feature values of the self-interference pattern at different defocus planes. (c) The relative variation of the four characteristic parameters at different defocuses planes.

**Supplementary Reference:**

1. Liu, D. *et al.* Three-dimensional controlled growth of monodisperse sub-50 nm heterogeneous nanocrystals. *Nat. Commun.* **7**, 10254 (2016).

2. P. B. Johnson and R. W. Christy. Optical Constant of the Nobel Metals. *Phys. L Re View B* **6**, 4370–4379 (1972).

3. Singer, W., Totzeck, M. & Gross, H. Handbook of Optical Systems, Volume 2, Physical Image Formation. *Handb. Opt. Syst.* (2005).

4. Schneider, P.-I. *et al.* Numerical optimization of the extraction efficiency of a quantum-dot based single-photon emitter into a single-mode fiber. *Opt. Express* **26**, 8479 (2018).

5. Liu, Y. *et al.* Amplified stimulated emission in upconversion nanoparticles for super-resolution nanoscopy. *Nature* **543**, 229–233 (2017).

6. Zhan, Q. *et al.* Achieving high-efficiency emission depletion nanoscopy by employing cross relaxation in upconversion nanoparticles. *Nat. Commun.* **8**, 1–11 (2017).

7. Lu, D. *et al.* Plasmon Enhancement Mechanism for the Upconversion Processes in NaYF 4 :Yb^3+^ ,Er^3+^ Nanoparticles: Maxwell versus Förster. *ACS Nano* **8**, 7780–7792 (2014).

8. Clarke, C. *et al.* Large-scale dewetting assembly of gold nanoparticles for plasmonic enhanced upconversion nanoparticles. *Nanoscale* **10**, 6270–6276 (2018).

9. Chen, C. *et al.* Multi-photon near-infrared emission saturation nanoscopy using upconversion nanoparticles. *Nat. Commun.* **9**, 4–9 (2018).

10. Dung, H. T., Knöll, L. & Welsch, D.-G. Intermolecular energy transfer in the presence of dispersing and absorbing media. *Phys. Rev. A* **65**, 043813 (2002).

11. Lu, D. *et al.* Plasmon Enhancement Mechanism for the Upconversion Processes in NaYF 4 :Yb^3+^ ,Er^3+^ Nanoparticles: Maxwell versus Förster. *ACS Nano* **8**, 7780–7792 (2014).

12. Chen, X.-W., Choy, W. C. H. & He, S. Efficient and Rigorous Modeling of Light Emission in Planar Multilayer Organic Light-Emitting Diodes. *J. Disp. Technol.* **3**, 110–117 (2007).

13. Wang, F. *et al.* Microscopic inspection and tracking of single upconversion nanoparticles in living cells. *Light Sci. Appl.* **7**, 18006–18007 (2018).

14. Tarantino, N. *et al.* TNF and IL-1 exhibit distinct ubiquitin requirements for inducing NEMO–IKK supramolecular structures. *J. Cell Biol.* **204**, 231–245 (2014).
