# Supplementary material for: Axial localization and tracking of self-interference nanoparticles by lateral point spread functions
Source: Nat Commun. 2021 Apr 1;12:2019. doi: 10.1038/s41467-021-22283-0 (PMC8016974; doi:10.1038/s41467-021-22283-0)
Supplement: Supplementary file 4 — Description of Additional Supplementary Files [file 41467_2021_22283_MOESM4_ESM.pdf]

Descriptions of Additional Supplementary Files

Supplementary Video 1

Video tracking of an upconversion nanoparticle (UCNP) with 33 nm diameter. The particle suspends inside a micro-chamber filled by glycerol solution (refractive index is 1.48). The imaging/sensing frame rate is 10Hz for compensating the reduced intensity from the UCNP by glycerol solution. During the measurement, the stage is periodically moving from 0 to 200 nm with the increment of 20 nm per frame to artificially induce large defocus drifts.
